# Machine Learning and Pharmacometrics for Prediction of Pharmacokinetic Data: Differences, Similarities and Challenges Illustrated with Rifampicin

**DOI:** 10.3390/pharmaceutics14081530

**Published:** 2022-07-22

**Authors:** Lina Keutzer, Huifang You, Ali Farnoud, Joakim Nyberg, Sebastian G. Wicha, Gareth Maher-Edwards, Georgios Vlasakakis, Gita Khalili Moghaddam, Elin M. Svensson, Michael P. Menden, Ulrika S. H. Simonsson, on behalf of the UNITE4TB Consortium

**Affiliations:** 1 Department of Pharmaceutical Biosciences, Uppsala University, 75124 Uppsala, Sweden; lina.keutzer@farmbio.uu.se (L.K.); huifang.you@farmbio.uu.se (H.Y.); 2 Computational Health Center, Helmholtz Munich, 85764 Neuherberg, Germany; alifarnod@gmail.com (A.F.); michael.menden@helmholtz-muenchen.de (M.P.M.); 3 Department of Pharmacy, Uppsala University, 75123 Uppsala, Sweden; joakim.nyberg@farmaci.uu.se (J.N.); elin.svensson@farmaci.uu.se (E.M.S.); 4 Department of Clinical Pharmacy, Institute of Pharmacy, University of Hamburg, 20146 Hamburg, Germany; sebastian.wicha@uni-hamburg.de; 5 Research, Clinical Pharmacology Modelling & Simulation, GlaxoSmithKline, London TW8 9GS, UK; gareth.x.edwards@gsk.com (G.M.-E.); georgios.x.vlasakakis@gsk.com (G.V.); gita.x.khalilimoghaddam@gsk.com (G.K.M.); 6 Department of Clinical Neurosciences, University of Cambridge, Cambridge CB2 0QQ, UK; 7 Department of Pharmacy, Radboud Institute of Health Sciences, Radboud University Medical Center, 6525 EZ Nijmegen, The Netherlands; 8 Department of Biology, Ludwig-Maximilian University Munich, 82152 Planegg-Martinsried, Germany; 9 German Center for Diabetes Research (DZD e.V.), 85764 Neuherberg, Germany

**Keywords:** machine learning, pharmacometrics, population pharmacokinetics, rifampicin, pharmacokinetics, simulation, feature selection

## Abstract

Pharmacometrics (PM) and machine learning (ML) are both valuable for drug development to characterize pharmacokinetics (PK) and pharmacodynamics (PD). Pharmacokinetic/pharmacodynamic (PKPD) analysis using PM provides mechanistic insight into biological processes but is time- and labor-intensive. In contrast, ML models are much quicker trained, but offer less mechanistic insights. The opportunity of using ML predictions of drug PK as input for a PKPD model could strongly accelerate analysis efforts. Here exemplified by rifampicin, a widely used antibiotic, we explore the ability of different ML algorithms to predict drug PK. Based on simulated data, we trained linear regressions (LASSO), Gradient Boosting Machines, XGBoost and Random Forest to predict the plasma concentration-time series and rifampicin area under the concentration-versus-time curve from 0–24 h (AUC_0–24h_) after repeated dosing. XGBoost performed best for prediction of the entire PK series (*R*^2^: 0.84, root mean square error (RMSE): 6.9 mg/L, mean absolute error (MAE): 4.0 mg/L) for the scenario with the largest data size. For AUC_0–24h_ prediction, LASSO showed the highest performance (*R*^2^: 0.97, RMSE: 29.1 h·mg/L, MAE: 18.8 h·mg/L). Increasing the number of plasma concentrations per patient (0, 2 or 6 concentrations per occasion) improved model performance. For example, for AUC_0–24h_ prediction using LASSO, the *R*^2^ was 0.41, 0.69 and 0.97 when using predictors only (no plasma concentrations), 2 or 6 plasma concentrations per occasion as input, respectively. Run times for the ML models ranged from 1.0 s to 8 min, while the run time for the PM model was more than 3 h. Furthermore, building a PM model is more time- and labor-intensive compared with ML. ML predictions of drug PK could thus be used as input into a PKPD model, enabling time-efficient analysis.

## 1. Introduction

Pharmacometrics (PM) and machine learning (ML) are promising approaches used in drug discovery and development, regulatory decision making and personalized medicine. In pharmacokinetic/pharmacodynamic (PKPD) analysis, pharmacokinetic (PK) data are commonly used as input to drive the exposure–response relationship, which can be both continuous drug concentrations or derived PK parameters (e.g., area under the concentration-time curve, AUC) [1,2]. When data are sparse, utilizing classical methodologies such as noncompartmental analysis (NCA) is not appropriate. In this case, PKPD modelling using the population approach is commonly applied. This requires the availability or development of a population PK model, which can be time- and labor-intensive. Growing access to big data in recent years has increased the interest in utilization of ML for predictions. ML, with its high computational efficiency, has significant potential and is starting to be used in drug development [3], but is not applied as much as in discovery yet [4].

**Figure 1:**
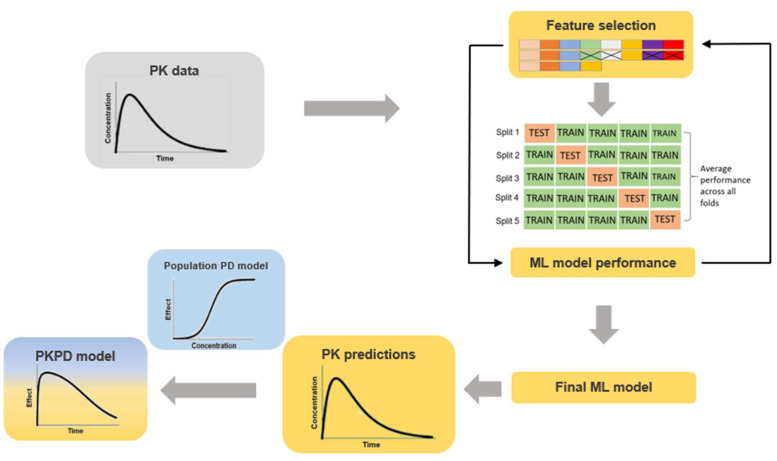
Overall proposed workflow. Blue panels indicate pharmacometrics and yellow machine learning.

While population PK modelling is a method very frequently used for drug PK predictions, ML is less commonly utilized. There are, however, a few examples in the literature where ML has been applied to predict PK data. Poynton et al. [5], for example, have evaluated the performance of several ML algorithms for prediction of remifentanil PK and compared the performance to population PK [5]. The authors show that an ensemble model integrating both an artificial neural network (ANN) and a population PK model best described remifentanil plasma concentration over time [5]. In addition, Woillard et al. [6,7] have shown that the XGBoost algorithm is appropriate for predictions of tacrolimus and mycophenolate mofetil exposure. Woillard et al. [6] and Poynton et al. [5] were some of the first authors to apply ML to PK datasets. While ML has the advantage of being fast and efficient, as well as being able to handle large datasets, PM models are based on biological mechanisms, contributing to mechanistical understanding, biological interpretability of the results and the potential to simulate in silico experiments from the model. ML carries an inherent risk of returning outputs that are not clinically relevant. Thus, this is another reason (other than time) that these two disciplines should work together. Due to the multiple challenges faced in PM and ML, there is a continuous interest in both communities to identify possible ways of combining expertise from both fields [4,8,9,10]. Attempts to combine PM and ML were made by others previously. Current work includes improved methods for covariate selection [8], as well as research focused on combining ML algorithms with the compartmental structure of PM models [11,12] aiming to improve efficiency in model development. One way of combining ML and PM could be to use PK predicted from an ML model as input for a pharmacometrics PKPD model, provided that the predictive performance is acceptable (illustrated in Figure 1). In order to evaluate whether ML could support PKPD modelling by fast and accurate prediction of PK data, the aim of this case study was to investigate the ability of different ML algorithms to accurately and precisely predict both plasma concentration over time and derived PK parameters (such as AUC), using rifampicin as an example. Rifampicin is an antibiotic commonly used to treat drug-susceptible tuberculosis as part of an approved four-drug regimen [13]. It is a drug known for its complex PK, including autoinduction of elimination, concentration-dependent nonlinear clearance and dose-dependent bioavailability [14,15,16], as well as a high inter-occasion variability (IOV) [17]. Nonlinear PK sometimes leads to a complex modelling process, increasing labor efforts and time needed. This is the key reason why we believe that using an ML algorithm can actually assist PM in being more efficient.

When merging both methods, it is critical to first of all understand similarities and differences between the two fields, to uncover gaps in either methodology and to establish a common terminology, enabling both communities to communicate with each other.

### 1.1. Pharmacometrics and Machine Learning

#### 1.1.1. Pharmacometrics

**Figure 2:**
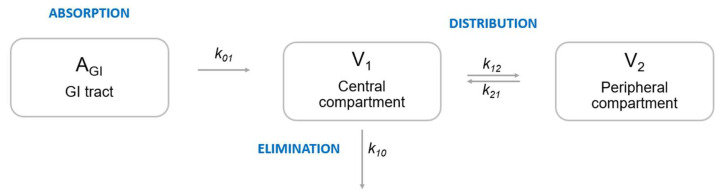
Illustration of a two-compartment pharmacokinetic model for a fictive drug. A_GI_, amount of drug in the gastrointestinal tract; *k*_01_, absorption rate constant; k_10_, elimination rate constant; *k*_12_, rate constant describing distribution from central to peripheral compartment; *k*_21_, rate constant describing distribution from peripheral to central compartment; V_1_, volume of central compartment (e.g., blood); V_2_, volume of peripheral compartment (e.g., brain tissue). Drug clearance is expressed as $k_{10}\times V_{1}$.

PM is the science of mathematical and statistical modelling with the aim of quantifying a drug’s PK, pharmacodynamics (PD), PKPD or disease progression. PM models can be utilized within the entire spectrum of drug development, from discovery all the way to life-cycle management and label updates [18,19,20,21,22,23,24,25]. According to the FDA, model-informed drug development (MIDD) is an integral component in the development of a drug [26]. A common method used in PM is nonlinear mixed effects (NLME) modelling, first defined by Lindstrom and Bates [27]. It describes simultaneous model fitting to PK or PD data from all individuals within a population. NLME models consist of fixed-effects parameters describing the population as a whole and random-effects parameters describing the variability within the population and in the individual [27,28]. A population can, e.g., be a group of patients, healthy volunteers, but even a set of in vitro or in vivo data. The aim of a PM analysis is typically to quantify both the variability in the PK or PD response between patients and within a patient, as well as to identify predictors (= covariates) informing about the source of variability (see Figure S1), which can be endogenous (data-driven) or exogenous (e.g., between study sites). NLME models describe (1) the general tendency within the population at hand, (2) the variability between different patients (inter-individual variability (IIV)), (3) variability within the same patient on different occasions (inter-occasion variability (IOV)), (4) the remaining residual unexplained variability (RUV) and (5) predictors descriptive of the variability between patients, termed covariates. NLME models are defined by ordinary differential equations (ODEs) and stochastic differential equations or analytical solutions [29,30]. They are usually expressed as compartmental models describing the absorption, distribution, metabolism and elimination of a drug, as illustrated in Figure 2. Since all data are fit simultaneously, model building with sparse or imbalanced data is possible [1,29,30]. Model building is performed in a step-wise manner and PM models contain biological or pharmacological mechanisms in their structure, thus allowing the derivation of parameters which are biologically sound and interpretable. The final model parameter estimates can be used to perform simulations of “what if” scenarios, answering research questions in order to inform future in vitro/in vivo experiments or clinical trials regarding their chances of success [31,32,33,34,35], which can greatly reduce the cost for an experiment or trial [36].

During and after model development, PM modellers utilize numerous diagnostics, both quantitative and/or graphical, in order to select the final model that best describes the observed data. For quantitative model comparison, modellers in the PM community commonly turn to the objective function value (OFV). The likelihood of the predictions to fit the data, i.e., the probability of the model parameters being able to describe the data, is estimated in a maximum likelihood parameter estimation, often using differential equation systems. The model parameters are estimated by minimizing the OFV, which is proportional to the −2*log likelihood that the model parameter values occur from the data. For comparison of nested models, the likelihood ratio test is used [37,38].

Graphical model evaluation is performed most commonly using visual predictive checks (VPCs) [39,40,41], basic goodness-of-fit (GOF) plots and individual plots [42,43]. The VPC is a tool to investigate the predictive performance of the model and allows for comparison between alternative models, evaluation of model fit and visualization of how the model could be improved [39]. The observed data are compared to data simulated from the model parameters. Commonly, the 90th percentile (sometimes lower) of the observed data is compared to the 95% (or lower) confidence interval (CI) of the simulated 90th percentile data. GOF plots include, for example, evaluation of population predictions or individual predictions versus observations (see Table 1 for details regarding terminology). In individual plots, individual observed data are compared to individual predictions. Lastly, precision in the parameter estimates, clinical relevance and scientific plausibility are considered during model evaluation.

#### 1.1.2. Machine Learning

Machine learning (ML) has been defined as “a field of statistical research for training computational algorithms that split, sort and transform a set of data to maximize the ability to classify, predict, cluster or discover patterns in a target dataset” [44]. ML is commonly divided into supervised, unsupervised and semi-supervised methods [45]. Supervised ML aims to predict human assigned labels or experimentally determined outputs based on independent variables, i.e., these models are trained to predict established and expected outcomes based on a loss function. Algorithms belonging to supervised ML models either solve regression and classification problems, e.g., logistic regression, neural networks, support vector machines and decision trees [46]. In contrast, unsupervised machine learning does not require prior knowledge regarding the outcomes, and aims to reveal unexpected patterns in the data, i.e., clustering [47]. Semi-supervised learning is a combination of using labelled and unlabelled data applied in situations where only parts of the data have been labelled [48]. ML has frequently been used in drug discovery and is now increasingly being applied to drug development. Applications in drug development include biomarker identification, prediction of clinical outcomes and planning of clinical trials [9,10,45,49,50,51].

Here, we focus on supervised ML, in which the dataset is separated into training, validation and test datasets. Common methodologies to split the dataset are either n-fold cross-validation or bootstrapping (for terminology, see Table 1). The training dataset is used for learning patterns in the data, while the validation dataset determines optimal parameterization, for instance, the number of trees in the decision tree, or epochs in a neural network. The loss function is calculated and optimized on the validation dataset to avoid under- or overfitting on the training dataset. Different methods for computation of the loss function exist, such as *L*1 (Least Absolute Deviations) (Equation (1)) or *L*2 (Least Square Errors) (Equation (2)).

(1) $$L1=\sum_{i=1}^{n}\left|Y_{observed}-Y_{predicted}\right|$$

(2) $$L2=\sum_{i=1}^{n}{\left(Y_{observed}-Y_{predicted}\right)}^{2}$$

In Equations (1) and (2), *L*1 indicates Lasso regression, *L*2 Ridge regression, *Y_observed_* observed data and *Y_predicted_* ML prediction. For estimating generalizability to predict novel data points [45], the test dataset is exclusively used for the final model evaluation, never for any training or parameterization [52,53,54]. Data leakage of the test dataset mostly leads to severe overestimation of the model’s predictive performance, i.e., overfitting [45].

#### 1.1.3. Terminology

Different terminologies are used in PM and ML, often describing either the same or a similar part of the analysis. While in PM one “builds” or “fits” a model, in ML a model is “trained”. Both terms essentially describe the process of developing a model, its structure and parameters of interest. When it comes to predictors used in the model, there is a difference between ML and PM. In ML, the word “features” is used to describe all input variables used to train the model in order to predict the desired outcome [54]. This could in drug development, for example, include time, dose and patient characteristics such as bodyweight, age or creatinine clearance. In PM, on the other hand, predictors are usually termed “covariates”, which are not directly comparable to features. Covariates describe predictors that aim to explain the sources of the PK and PD variability between patients (IIV). Since certain variables such as time and dose are normally already part of the structural model, they are not considered covariates [55]. Only the predictors explaining variability between patients in addition to the variables already included in the structural model are considered covariates, which would in this example be bodyweight, age or creatinine clearance. In this work, we use the word “predictor” as an umbrella term for both feature and covariate. In PM, only scientifically plausible and clinically relevant predictors should be included in the model, which are identified in a preselection step conducted by an expert. This can also be beneficial for ML models; however, if the dataset is large enough, preselection of predictors is not necessary. ML can then be used to identify unexpected associations in a data-driven manner.

The parameters of a PM model are often not comparable to those in ML. In PM, parameters are descriptive of PK and PD processes, such as drug clearance, distribution volume or drug absorption, as well as drug effect (e.g., the maximal drug effect (E_max_) or the drug’s potency, such as EC_50_). ML parameters, however, are more mathematical and from a biological point of view less interpretable. Parameters are configuration variables of the model and are estimated during the model training process enabling predictions from the final model. They are determined automatically and include, for example, weights, coefficients and support vectors. Hyperparameters, on the other hand, are variables defined by the modeller as they cannot be estimated from the data, but are tuned during the learning process [56], such as the regularization parameter λ and the number of trees *k* in eXtreme Gradient Boosting (XGBoost) [57]. Determination of hyperparameters is called “hyperparameter tuning” and is often achieved by testing different hyperparameters and then choosing the ones providing the best model fit [58,59]; however, the process can also be automated [59].

As mentioned above, in supervised ML the data are split into a “training dataset” used for learning general rules, a “validation dataset” for internal validation and a “test dataset” for unbiased evaluation using cross-validation [52,53,54]. Separating the data is not common practice in pharmacometrics model building, where, instead, all available data are used for model building and only external validation is performed with new data.

**Figure 3:**
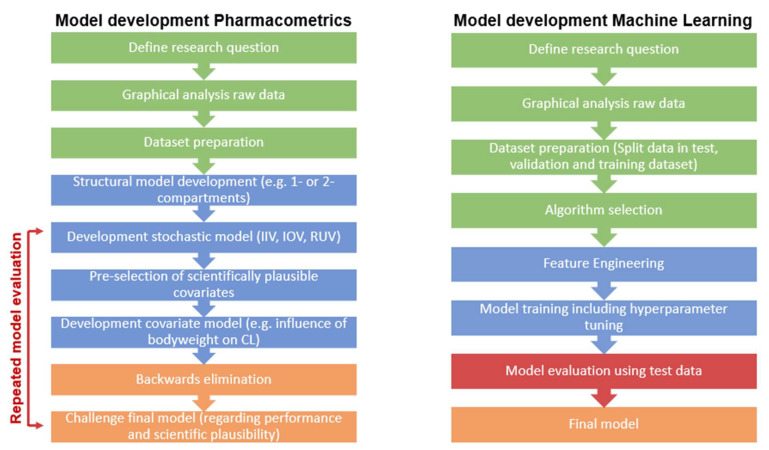
Comparison of the general model development workflow between pharmacometrics and machine learning. The different colors represent different steps of model development. Green: data preparation, blue: model building, red: model evaluation, orange: finalizing the model.

The overall workflows for both PM and ML are illustrated in Figure 3.

Table 1 provides a comprehensive overview of differences and similarities in terminology used by the PM and ML community.

**Table 1:** Overview of terminology commonly used by the pharmacometrics and/or machine learning community.

Term		Description
PM	ML	
Covariates	Features	Both terms describe predictors. Features are all input variables used to train a model. Covariates are predictors explaining variability between patients in addition to the variables already included in the structural pharmacometrics model.
Objective function value (OFV)	Loss	The OFV is one of the main metrics for model evaluation in pharmacometrics model building. It is proportional to −2*log likelihood that the model parameter values occur from the data [37,38]. In ML, the loss is used as a goodness of fit. It represents the distance between predictions and observations which can be computed in different ways, such as *L*1, *L*2 or MAPE.
Build/Fit a model	Train a model	Both terms define the process of developing a model by determining model parameters that describe the input data in order to reach a predefined objective.
Validation dataset	Validation dataset	In PM, the term validation dataset is often used for external validation. In ML, the term is commonly used for the data that are held back for internal validation to evaluate model performance during training.
Overparameterization	Overfitting	In PM, a model can be overparameterized, meaning too many parameters are estimated in relation to the amount of information, leading to minimization issues. Overfitting in ML describes a phenomenon where the model has been trained to fit the training data too well. The model is forced to predict in a very narrow direction, which may result in poor predictive ability.
Model parameters	Model parameters	Even though both communities use the same term, model parameters in PM are different from parameters in ML. Model parameters in PM describe biological or pharmacological processes, such as drug clearance, drug distribution volume or rate of absorption. These parameters are directly interpretable. In ML, on the other hand, model parameters are mathematical parameters learnt during the model training process and are part of the final model describing the data. They do not provide biological interpretation in the first instance at least.
Model averaging	Ensemble model	An ensemble model combines multiple ML algorithms, which in most cases leads to better predictive performance compared to single algorithms [60]. There is a similar method used in PM called model averaging [61], where several models are combined using weights determined by their individual fit to the data.
Shrinkage	Shrinkage	The term “shrinkage” has a different meaning in the PM and ML communities. In PM, shrinkage describes overparameterization, where 0 indicates very informative data and no overfit, and 1 uninformative data and overfitting. In ML, shrinkage methods in different ML models reduce the possibility of overfitting or underfitting by providing a trade-off between bias and variance.
Bootstrapping	Bootstrapping	Describes a random resampling method with replacement. In PM, it is used during model development and evaluation for estimation of the model performance. In ML, bootstrapping is part of some algorithms, such as XGBoost or Random Forest, and is also used to estimate the model’s predictive performance.
Cross-validation	Cross-validation	In PM, cross-validation is used occasionally, for example, in covariate selection procedures in order to assess the true alpha error. In ML, cross-validation is commonly applied to prevent overfitting and to obtain robust predictions. Cross-validation describes the process of splitting the data into a training dataset and a test dataset. The training dataset is used for model development and the test dataset for external model evaluation. In n-fold cross-validation, the data are split into n non-overlapping subsets, where n − 1 subsets are used for training and the left-out subset for evaluation. This procedure is repeated until all subsets have been used for model evaluation. Model performance is then computed across all test sets [45].
-	Holdout/test dataset	Describes the test/unseen dataset used for external validation. It is of great importance that the holdout/test data is not used for model training or hyperparameter tuning in order not to overestimate the model’s predictive performance [45].
-	Oversampling/Upsampling	Oversampling is an approach used to deal with highly imbalanced data. Data in areas with sparse data are resampled or synthesized using different methods, for example, Synthetic Minority Oversampling Technique (SMOTE) [62].
Empirical Bayes Estimates (EBEs)	Bayesian optimization	EBEs in PM are the model parameter estimates for an individual, estimated based on the final model parameters as well as observed data using Bayesian estimation [63]. In artificial intelligence (AI), Bayesian optimization is used to tune artificial neural networks (ANNs), particularly in deep learning.
Typical value	Typical value	The typical value in PM is the most likely parameter estimate for the whole population given a set of covariates. It could, e.g., be the drug clearance estimate that best summarizes the clearance of the whole population. In ML, the typical value in unsupervised learning, for example, is the center of a cluster (e.g., k-means).
Inter-individual variability (IIV)	-	Variability between individuals in a population. Describes the difference between typical and individual PK parameters. Often assumed to be log-normally distributed.
Inter-occasion variability (IOV)	-	Variability within an individual on different occasions (e.g., sampling or dosing occasions). Often assumed to be log-normally distributed.
Residual error variability (RUV)	-	Remaining random unexplained variability. Describes the difference between individual prediction and observed value.
Population prediction	-	The population prediction is the most likely representation of the population given a set of covariates.
Individual prediction	-	Predictions for an individual using the population estimates in combination with the observed data for this individual, computed in a Bayesian posthoc step.

*L*1, least absolute deviations; *L*2, least absolute errors; MAPE, mean absolute prediction error; ML, machine learning; PM, pharmacometrics.

## 2. Materials and Methods

### 2.1. Data

Rifampicin PK data were simulated from a previously published population PK model describing rifampicin plasma concentrations over time in tuberculosis patients [15], which has been shown to perform best compared to other population PK models for rifampicin and has undergone external validation [64]. The population PK model consists of a 1-compartment distribution model with nonlinear elimination (described as a Michaelis–Menten equation), autoinduction of elimination and dose-dependent bioavailability [15]. The simulated scenario was as in the original HIGHRIF1 clinical phase 2 trial [65] in order to create clinically relevant data. PK in 83 individuals following 10, 20, 25, 30, 35 or 40 mg/kg oral rifampicin once a day for 2 weeks was simulated. The dose group was randomly assigned to a simulated individual. The simulated individual’s bodyweight defined the actual dose. The dose was then rounded to the next 150 mg increment, due to availability of tablet strength. In total, the 13 doses, which are 450, 600, 900, 1050, 1200, 1350, 1500, 1650, 1800, 1950, 2250, 2400, 2550 and 2700 mg, were randomly assigned to 8, 9, 1, 7, 8, 3, 17, 2, 13, 5, 3, 4, 1 and 2 individuals, respectively. IIV, IOV and RUV were included in the simulations. PK sampling time-points were at pre-dose and 0.5, 1, 1.5, 2, 3, 4, 6, 8, 12 and 24 h after dose at two different sampling occasions (day 7 and 14 of treatment). Patient covariates were randomly sampled from the parametric covariate distribution in the HIGHRIF1 trial [65], taking into account the correlation between bodyweight (WT), fat-free mass (FFM) and gender, as described previously [17]. The covariates were sampled from either a truncated normal distribution (WT, FFM, age, body height (HT)) or a binomial distribution (gender, HIV-coinfection, race). Body mass index (BMI) was calculated from WT and HT.

Predictors included in the simulations to create the dataset were time after dose (TAD), treatment week (OCC) and dose, which are part of the structural PM model, as well as the covariate FFM. In total, 1826 (83 patients × 2 occasions × 11 samples per occasion) rifampicin plasma concentrations were simulated. The simulated rifampicin concentrations and the covariates included in the simulation are considered to be the true observed concentrations and predictors, respectively. The final simulated dataset is provided in Supplementary Materials Data S1.

### 2.2. ML Model Training

In this work, we evaluate ML model performance for prediction of PK data using rifampicin as an example drug. Since either a rifampicin plasma concentration-time series or exposure indices can be used as input for a PKPD model, it is of interest to investigate the predictive ability of ML for either outcome. As an exposure index, the area under the plasma concentration-time curve from 0 to 24 h (AUC_0–24h_) was chosen here, since the AUC_0–24h_/MIC has been shown to be the best predictor of rifampicin efficacy [66]. The individual AUC_0–24h_ values were calculated using noncompartmental analysis (NCA) based on rich simulated profiles (20 observations per sampling occasion). For AUC_0–24h_ calculation, the trapezoidal rule implemented in the pmxTools R package [67] was utilized. The derived AUC_0–24h_ values were considered the true values.

For ML model training, features included in the training dataset were TAD, dose, OCC, BMI, age, gender, race, WT, HT, HIV co-infection and FFM. The target was either the rifampicin plasma concentration or the rifampicin AUC_0–24h_. From the whole dataset, 5 datasets each containing 80% of the data for training and 20% for testing were created using patient identifier (ID) as a grouping variable, enabling 5-fold cross-validation. When splitting the data, it was ensured that each of the 5 test datasets contained different IDs, making sure that every ID was left out once, thus avoiding overlapping and bias. The test set was solely used to evaluate final performance, never for any training or fitting parameters of any model. The training dataset was again split in 80% training and 20% validation for 5-fold internal cross-validation. Utilizing the training datasets as input, different ML algorithms were trained for prediction of rifampicin PK. The predictive performance for each of the 5 test datasets was averaged in order to compute the overall predictive performance.

The current study deals with a regression problem; therefore, different supervised ML algorithms included in the SuperLearner R package [68] were tested. The three top-performing algorithms were chosen for further evaluation, which were GBM, XGBoost and Random Forest. In addition to these three nonlinear models, a linear model (LASSO) was evaluated for comparison.

The algorithms were optimized by testing different model parameters (hyperparameter tuning). Hyperparameter tuning was performed in a grid search for LASSO, GBM and Random Forest, where all possible parameter combinations were explored. For XGBoost, a sequential, heuristic search was performed due to very long run times for the full grid search. The ranges of investigated hyperparameters of the final models are presented in Table S1.

For comparison of run times, as well as creation of a VPC, the population PK model [15] was re-estimated in NONMEM [37] using the simulated dataset.

### 2.3. Feature Ranking

GBM, XGBoost, Random Forest and LASSO were compared based on their capability to identify features. Since the dataset was created by simulating from the population PK model, including TAD, OCC, dose and FFM, which were previously shown to influence rifampicin PK [15], the data include a correlation between rifampicin plasma concentrations and these predictors. Other predictors included in the dataset, which were not used in the simulation of the plasma concentrations, can therefore be considered noise. In contrast to a real-world dataset, where the true predictors are unknown, in this simulation-based study the true predictors are known. In this work, we explored whether the four different algorithms were able to identify these true predictors, i.e., TAD, OCC, dose and FFM. Evaluated predictors were TAD, dose, OCC, BMI, age, gender, race, WT, HT, HIV co-infection and FFM. The machine learning model evaluates all features and assigns weights to them in the training process, which will determine the prediction of the outcome variable.

The importance score of GBM is based on calculating the amount by which each feature’s split point improves the efficiency in a single decision tree. The importance scores are averaged across all decision trees. Therefore, the greater the improvement efficiency measure of a feature on the split point (closer to the root node), the greater the weight. This means that the more the promotion tree is selected, the higher the feature importance. XGBoost, as the implemented method of GBM, has the same algorithms in feature ranking, but uses a more regularized model formalization to control over-fitting which would affect the importance scores. Random Forest calculates importance scores by evaluating the decrease in node impurity weighted by the probability of reaching that node. LASSO adds the *L*1 norm of the coefficient as a penalty term to the loss function. Since the regularization term is non-zero, the coefficients corresponding to less important features are therefore discarded.

### 2.4. PK Predictions

The amount of available clinical PK data is very different across the several stages of drug development and thus an additional objective was to explore ML model performance across varying data sizes. To assess the amount of information required by the ML algorithms to predict well, scenarios including varying numbers of observed rifampicin concentrations as input variables in addition to the weighted features included in the model (TAD, dose, OCC, BMI, age, gender, race, WT, HT, HIV co-infection and FFM) were investigated (see Table 2). Based on this input, a full plasma concentration-time series (at pre-dose and 0.5, 1, 1.5, 2, 3, 4, 6, 8, 12 and 24 h post-dose) and the AUC_0–24h_ at treatment days 7 and 14 were predicted (see Table 2). As an example, in scenario 2 (Table 2), the abovementioned features, as well as 2 observed rifampicin plasma concentrations at 2 and 4 h post-dose are used as input variables to the model to predict the rifampicin plasma concentration at pre-dose and 0.5, 1, 1.5, 2, 3, 4, 6, 8, 12 and 24 h post-dose, i.e., a full pharmacokinetic profile.

### 2.5. Model Evaluation

Model performance was evaluated using the *R*^2^ (Equation (3)) between observations and predictions, the root mean square error (*RMSE*) describing precision (Equation (4)) and the mean absolute error (*MAE*) describing bias (Equation (5)).

(3) $$R^{2}=1-\frac{SS_{res}}{SS_{tot}}$$

In Equation (3), *SS_res_* are the squared residuals reflecting the fit between observed and predicted value and *SS_tot_* are the total sum of squares, reflecting the total variance. *SS_res_* is defined as

$$SS_{res}=\sum {\left({\mathrm{Observed}}_{i}-Predicted_{i}\right)}^{2}$$

where *Observed_i_* is the individual plasma concentration or AUC_0–24h_ value simulated from the population PK model, and *Predicted_i_* is the individual ML model-based prediction.

*SS_tot_* is defined as

$$SS_{tot}=\sum {\left({\mathrm{Observed}}_{i}-\mathrm{mean}\left(Observed\right))\right)}^{2}$$

where *Observed_i_* is the individual plasma concentration or AUC_0–24h_ value simulated from the population PK model.

(4) $$RMSE=\sqrt{\frac{1}{n}\sum_{i=1}^{n}{\left({\mathrm{Observed}}_{i}-Predicted_{i}\right)}^{2}}$$

(5) $$MAE=\frac{1}{n}\;\sum_{i=1}^{n}|{\mathrm{Observed}}_{i}-Predicted_{i}|$$

In Equations (4) and (5), RMSE stands for root mean squared error describing precision, *MAE* stands for mean absolute error describing bias, ${\mathrm{Observed}}_{i}$ is the individual plasma concentration or AUC_0–24h_ value simulated from the population PK model and *Predicted_i_* is the individual ML model-based prediction.

### 2.6. Software

For simulation of rifampicin plasma concentrations, NONMEM version 7.3.0 (Icon Development Solutions, Hanover, MD, USA) [37] assisted by PsN version 5.0.0 (Department of Pharmacy, Uppsala University, Uppsala, Sweden) [43] was used. Dataset manipulation, data visualization and ML model training was performed in R statistical software version 4.0.3 (R Foundation for Statistical Computing, Vienna, Austria) [69]. The computations were performed on resources provided by the Swedish National Infrastructure for Computing (SNIC) through Uppsala Multidisciplinary Center for Advanced Computational Science (UPPMAX).

## 3. Results

### 3.1. Feature Ranking

**Figure 4:**
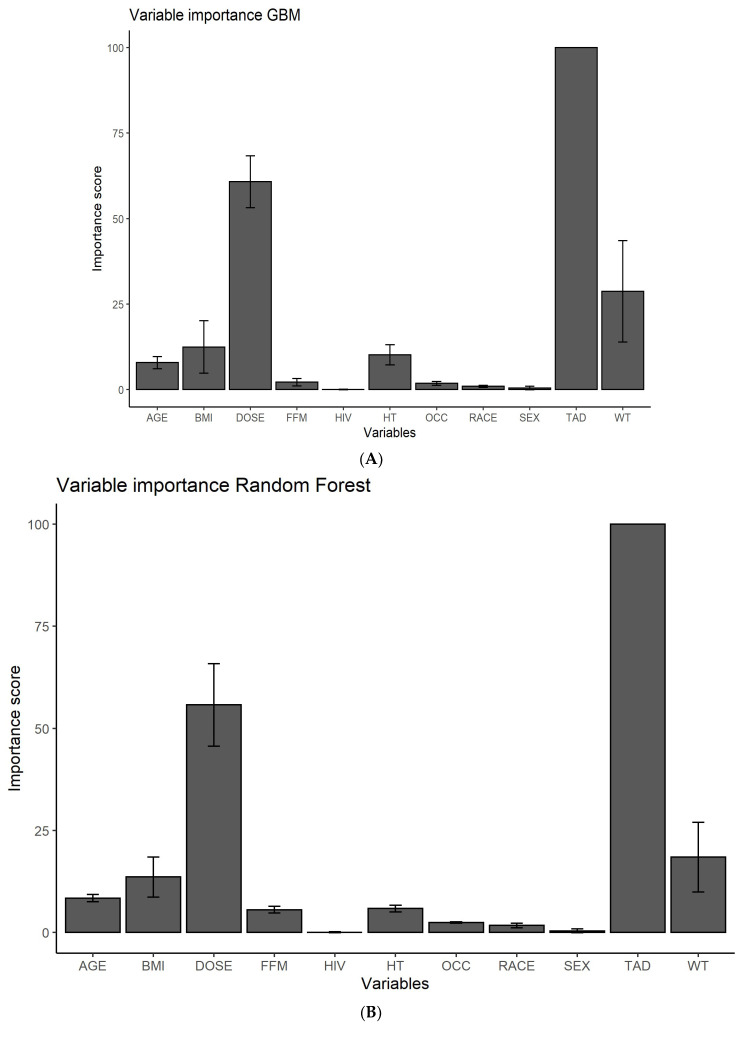

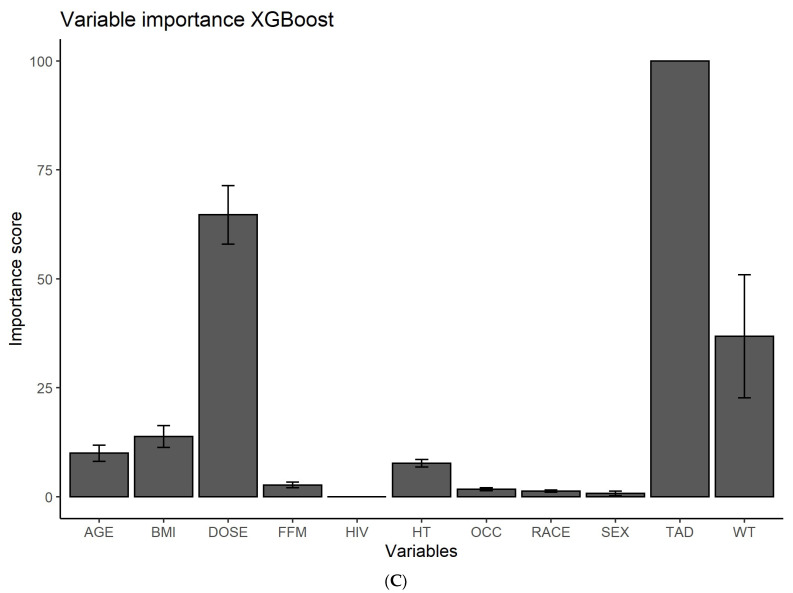
Importance scores for evaluated features shown for the different machine learning algorithms. (**A**) GBM, (**B**) Random Forest and (**C**) XGBoost using features only (scenario 1) as input for prediction of plasma concentration versus time. The error bars represent the standard deviation. AGE, age (years); BMI, body mass index (kg/m^2^); DOSE, daily rifampicin dose (mg); FFM, fat-free mass (kg); HIV, HIV-coinfection; HT, body height (cm); OCC, treatment week; RACE, race; SEX, gender; TAD, time after dose (h); WT, bodyweight (kg).

In scenario 1, i.e., predicting a rifampicin plasma concentration-time series based on features only, XGBoost, Random Forest and GBM selected TAD and dose as the most important predictors as expected, since these were included in the creation of the data. Instead of the true covariate FFM, all three algorithms selected WT and BMI as the third and fourth most important predictors, which can be explained by the direct correlation between WT, BMI and FFM. The linear LASSO algorithm showed poor feature selection performance, which was not better than random. Therefore, feature importance was not evaluated for LASSO. The importance score of each feature for the different ML algorithms (scenario 1) is presented in Figure 4. The feature importance for the five other scenarios is graphically presented in Figure S2. 

### 3.2. Predictions of Rifampicin Plasma Concentration over Time

XGBoost, Random Forest, GBM and LASSO were trained and optimized. The final, well-trained models were used to predict rifampicin plasma concentration over time (11 time-points per sampling occasion) using the features in the test dataset as input. The final model predictions and run times are presented in Table 3. For all algorithms, the run time was shorter (range across all algorithms: 1.0 (scenario 5 in Lasso)–508.7 s (scenario 3 in Random Forest)) compared to the population PK model (11,479 s) using 1 core of the UPPMAX cluster.

**Figure 5:**
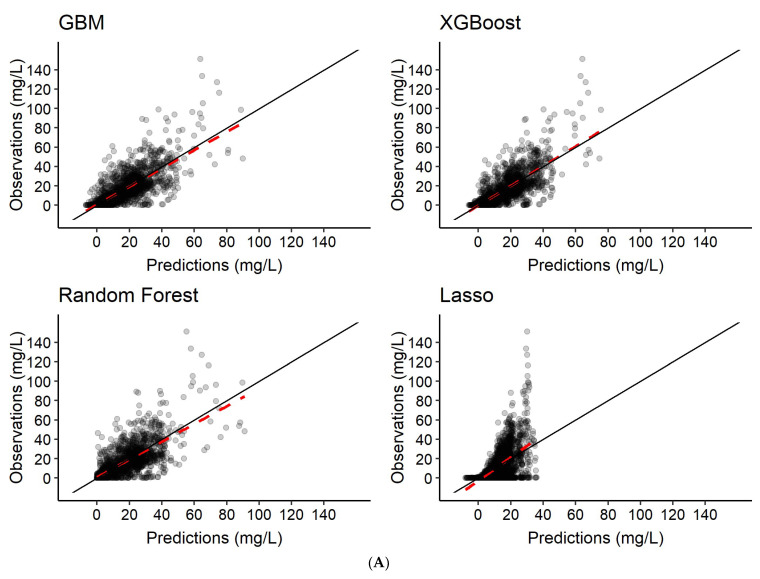

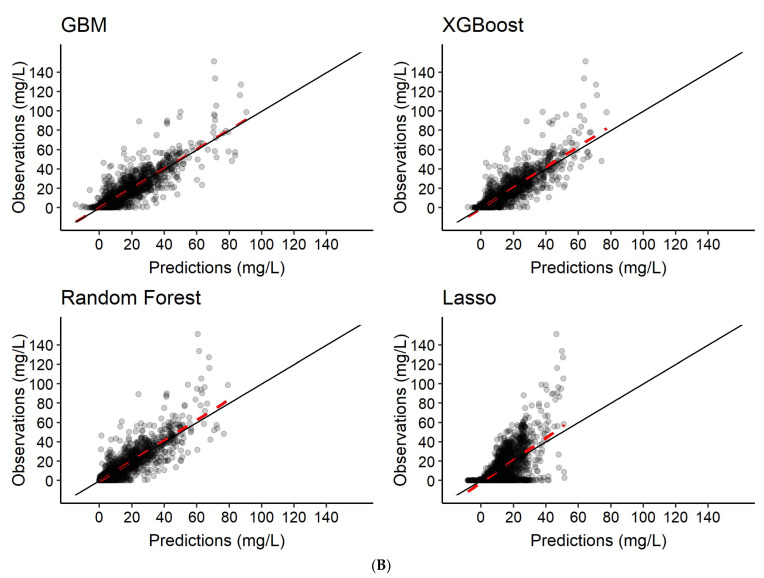

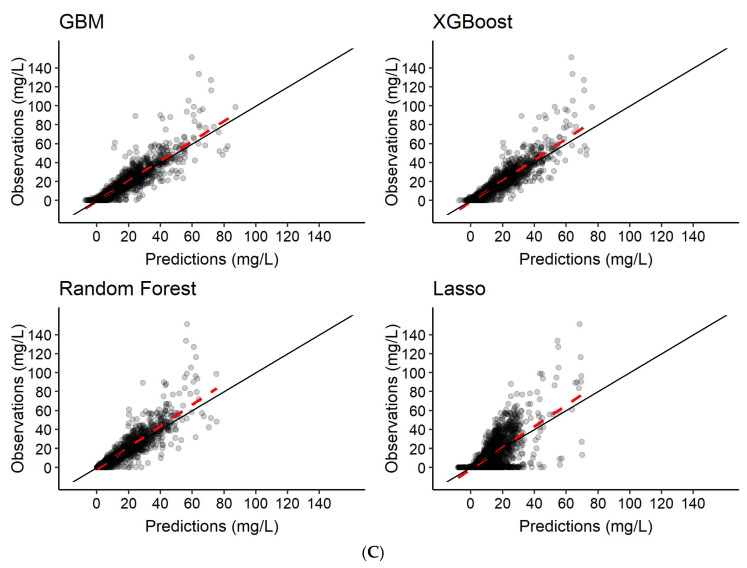
Predictions of rifampicin plasma concentration-time series from the different ML algorithms compared to the simulations from the population PK model, considered to be observations in this study. Panel (**A**) is the scenario where the model was trained to predict the rifampicin plasma concentration-time series using features only as input. In panel (**B**), the models were trained to predict the rifampicin plasma concentration-time series based on features and 2 plasma concentrations at time-points 2 and 4 h post-dose at days 7 and 14. In panel (**C**), the models were trained to predict the rifampicin plasma concentration-time series based on features and 6 plasma concentrations at time-points 0.5, 1, 2, 4, 8 and 24 h post-dose at days 7 and 14. The red dashed line represents a trendline through the data. The black solid line is the line of identity, indicating 100% agreement between true and predicted values.

**Figure 6:**
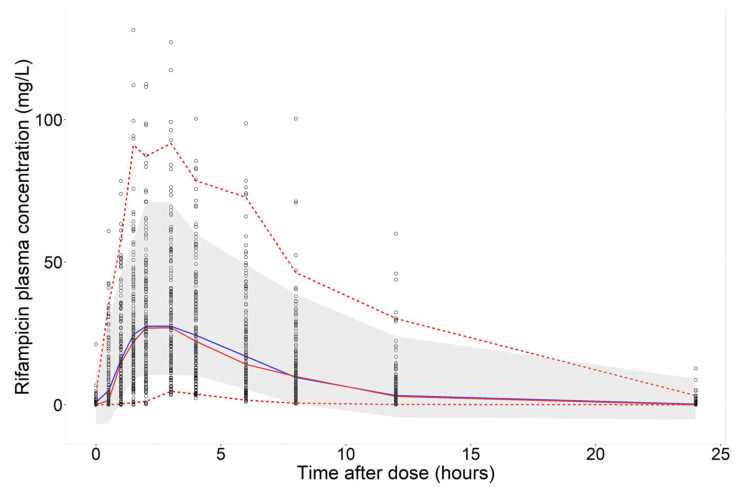
Prediction interval visual predictive check for the best-performing model (XGBoost) trained using 6 plasma concentrations as input (scenario 3) shown for the whole population. Open circles are the rifampicin plasma concentrations simulated from the population PK model, considered to be observed data in this study. The shaded area is the 95th prediction interval of the machine learning model predictions (XGBoost) and the solid blue line is the median of the model predictions. The upper and lower red dashed lines are the 97.5th and 2.5th percentiles of the observed data, respectively, and the solid red line is the median of the observed data.

**Figure 7:**
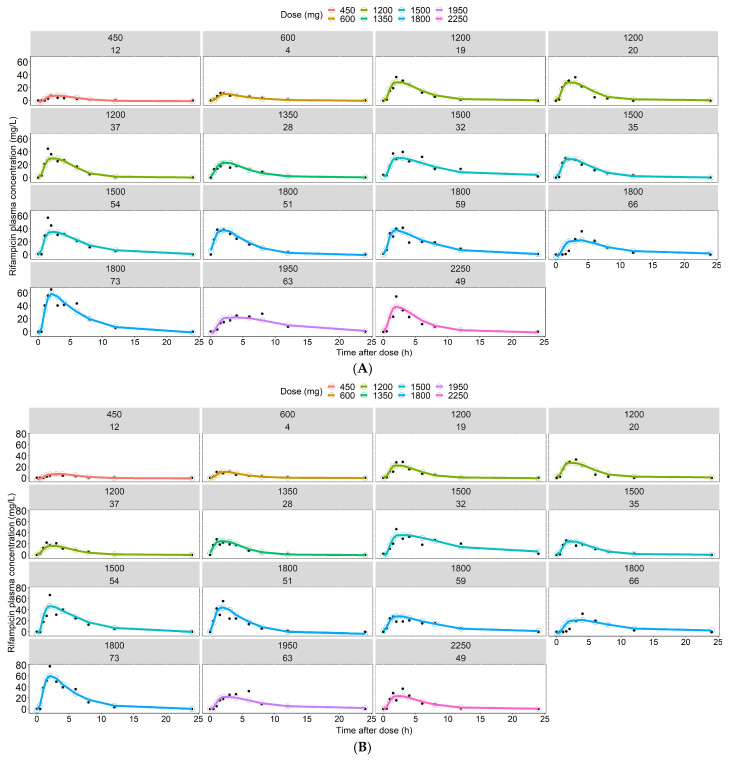
Individual rifampicin plasma concentrations predicted from the XGBoost model (solid line and open circles) compared to the concentrations simulated from the population PK model, considered to be observations in this study (black closed circles) shown for scenario 3 (features and 6 plasma concentrations used for prediction) for 15 randomly selected IDs. Panel (**A**) represents the predictions for each individual in the test dataset at day 7. Panel (**B**) represents the predictions for each individual in the test dataset at day 14. The different colors indicate the different daily rifampicin doses.

**Figure 8:**
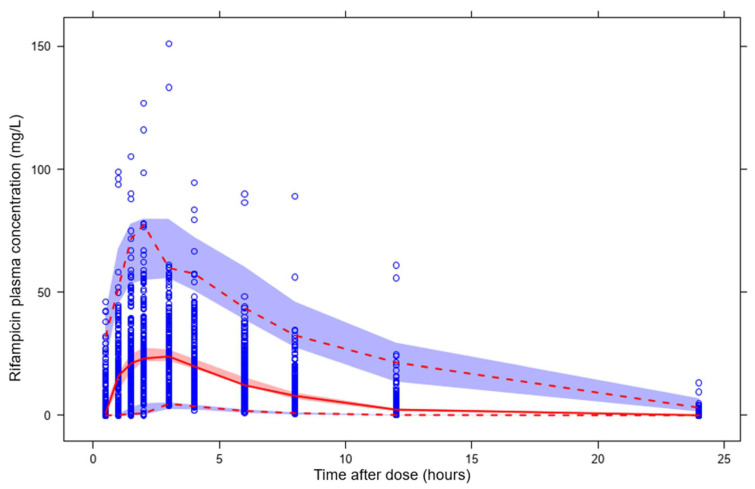
Visual predictive check for the re-estimated population PK model based on the simulated data. Open blue circles are the rifampicin plasma concentrations simulated from the population PK model, considered to be observed data in this study. The upper and lower dashed lines are the 95th and 5th percentiles of the observed data, respectively, and the solid line is the median of the observed data. The shaded areas (top to bottom) are the 95% confidence intervals of the 95th (blue shaded area), median (red shaded area) and 5th (blue shaded area) percentiles of the simulated data.

With regards to prediction of plasma concentration over time using features as input only, XGBoost showed the highest *R*^2^ (0.60) and precision (RMSE: 10.6 mg/L). GBM had an *R*^2^ of 0.57 and RMSE of 10.9 mg/L and Random Forest had an *R*^2^ of 0.54 and RMSE of 11.3 mg/L. The linear model LASSO had an *R*^2^ of 0.25. Using 2 rifampicin plasma concentrations per sampling occasion as input for prediction of the whole time series (11 time-points per sampling occasion) substantially improved model performance compared to features only. In this scenario, XGBoost and GBM had the highest predictive performance with an *R*^2^ of 0.76, closely followed by Random Forest with an *R*^2^ value of 0.75. The use of 6 rifampicin plasma concentrations led to the best predictive performance in all algorithms. XGBoost had the highest *R*^2^ (0.84) and precision (RMSE: 6.9 mg/L). Random Forest and GBM showed comparable performances, with *R*^2^ values of 0.82 and 0.83, respectively. LASSO exhibited poor performance (*R*^2^: 0.39). Model performance across all algorithms and scenarios is summarized in Table 3. The results clearly show that increasing the amount of data per simulated patient improves the predictive performance of all four ML algorithms, as shown in Table 3 and Figure 5. The prediction interval-based VPC for the best performing algorithm (XGBoost) (Figure 6) shows accurate prediction of the median but underprediction of the true variability in the population. The rifampicin concentrations simulated from the population PK model, considered to be observations in this study, for 15 randomly selected IDs in the test dataset and the model predictions from the best-performing ML model (XGBoost) were compared across the dose groups, which are presented in Figure 7 and Figure S3. Even though XGBoost showed the best predictive performance in scenarios 1, 2 and 3 (see Table 3), all three nonlinear algorithms exhibited acceptable predictive performance, considering the small dataset. A VPC for the re-estimated population PK model using the simulated data is shown in VPC (Figure 8).

Using the best performing model (XGBoost) and 6 rifampicin concentrations as input (scenario 3), the 95% prediction interval (PI) was −0.2–62.4 mg/L (median: 10.9 mg/L) compared to the data simulated from the population PK model with a 95% PI of 0–69.4 mg/L (median: 10.2 mg/L). Imprecision and bias were comparable between treatment week 1 (RMSE: 7.1 mg/L, MAE: 3.9 mg/L) and week 2 (RMSE: 6.6 mg/L, MAE: 4.1 mg/L), indicating that the ML model can identify the difference in exposure between week 1 and week 2 caused by rifampicin autoinduction.

The R code for the final models is provided in Supplementary Materials Text S2.

### 3.3. Predictions of Rifampicin AUC_0–24h_

**Figure 9:**
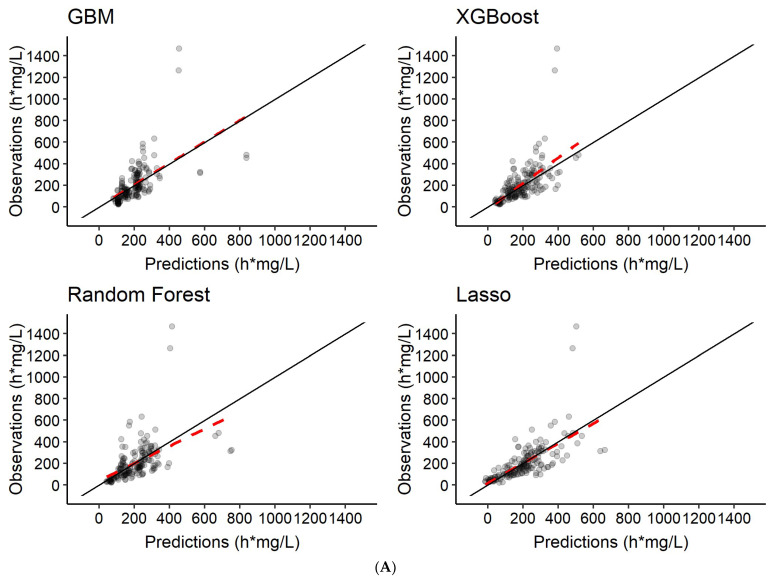

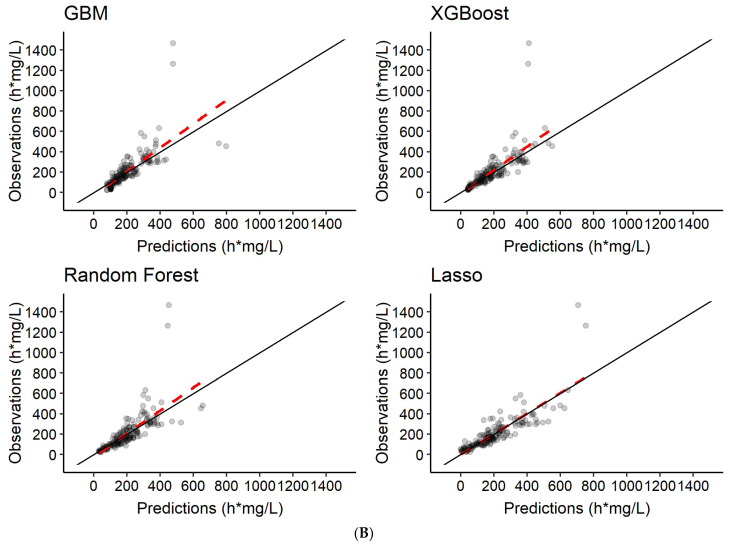

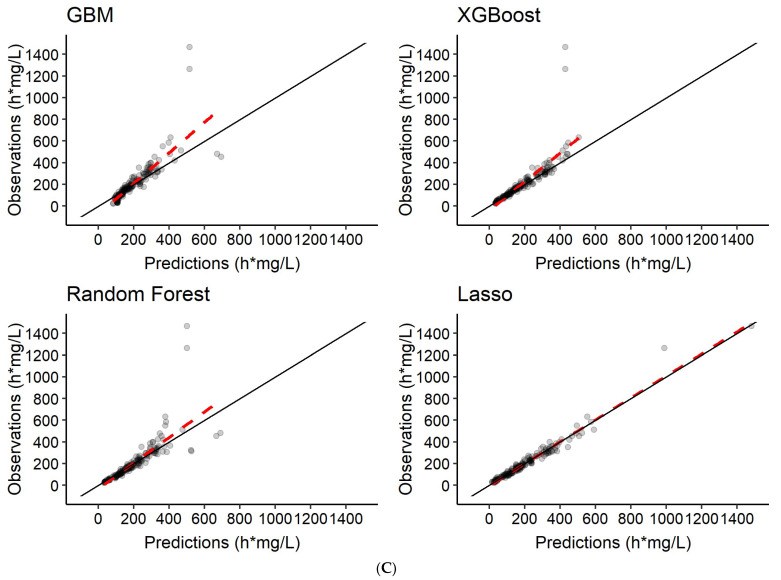
Predictions of rifampicin AUC_0–24h_ at days 7 and 14 from the different ML algorithms compared to the NCA derived AUC_0–24h_, considered to be observations in this study. Panel (**A**) is the scenario where the model was trained using features only as input. In panel (**B**), the models were trained to predict rifampicin AUC_0–24h_ based on features and 2 plasma concentrations at time-points 2 h and 4 h post-dose at days 7 and 14. In panel (**C**), the models were trained to predict rifampicin AUC_0–24h_ based on features and 6 plasma concentrations at time-points 0.5 h, 1 h, 2 h, 4 h, 8 h and 24 h post-dose at days 7 and 14. The red dashed line represents a trendline through the data. The black solid line is the line of identity, indicating 100% agreement between true and predicted values.

The different ML models were trained to predict rifampicin AUC_0–24h_ using varying plasma concentrations of rifampicin as input (see Table 2). The *R*^2^, imprecision (RMSE), bias (MAE) and run time for each scenario are summarized in Table 4. Graphical exploration revealed good performance across all four algorithms (Figure 9), but LASSO was superior in regard to precision and accuracy (Table 4).

## 4. Discussion

When comparing PM and ML methodology in drug development in terms of physiological plausibility and clinical relevance, the difference that probably stands out the most is that PKPD models are based on statistical models as well as underlying biological mechanisms where the models can vary from compartmental models to full mechanistic as in physiology-based pharmacokinetic models, while ML is often a purely data-driven approach, though not in all cases. In PM, the parameters are directly biologically interpretable, which can aid in identifying underlying mechanisms. ML parameters, however, are mathematically interpretable albeit to a lesser extent from a biological perspective. There are, however, some artificial intelligence (AI) methodologies, such as causal AI, that provide a better interpretability. In PM, the previous mechanistic knowledge that is used as input can also introduce a bias into the analysis if imputed inappropriately, which is avoided in ML. On the other hand, the previous knowledge used in PM models is crucial for analysis of sparse datasets. There are also differences in the model-building process. When already-available ML models are used, the modeller is less involved in the model building but facilitates the selection of an appropriate algorithm, hyperparameter tuning and model evaluation. In PM, a model is manually built in an iterative manner, constantly evaluating the model’s performance at each step, based on which the modeller takes the decision on how to proceed (Figure 3). The process is thus very time- and labor-intensive, and can lead to different modellers developing slightly different models based on the same dataset. This way of developing a model ensures that modelling is guided by a physiological intent, i.e., it is easily understandable if a PK profile follows a one- or a two-compartment model structure and how that potentially relates to the distribution of the drug in the body and site of action. ML cannot account for that in the first instance at least, but is a faster, more time- and labor-efficient method compared to PM. Challenges with purely data-driven ML algorithms certainly lie in the need for rich data, as well as the “Garbage in–Garbage out” problem, which describes the need for high-quality and diverse data in order to build robust models [45]. One of the major advantages of PM modelling is the ability to perform simulations of different scenarios that were not part of the data used for model building. An example is clinical trial simulations where a drug’s PK and/or effect is predicted in a virtual population for different scenarios, for example, to support dose selection for a future clinical trial or for prediction of optimal doses in renal impairment patients or pediatric populations [70]. While this can also be achieved using ML, e.g., with reinforcement methodologies, larger amounts of data are needed to train robust models.

In this work, we have investigated the identification of key features (Figure 4), described the variability in the PK predictions (Figure 6) and predicted PK using time as a continuous variable (Table 3, Figure 6 and Figure 7).

Accurate selection of informative predictors is crucial both to obtain a high model performance and for clinical use, as they inform about potential differences in dose adjustments in special populations. This work suggests that all three nonlinear algorithms are able to correctly identify TAD and dose as the most important predictors. Since the linear LASSO algorithm performed very poorly, feature selection was not evaluated. Despite the correct identification of TAD and dose, none of the nonlinear algorithms selected FFM in third place, which is the true covariate. All three algorithms, however, selected WT and BMI as third and fourth most important, which are directly correlated with FFM. We hypothesize that WT was assigned a high importance instead of FFM due to the high correlation between the two features. The ML model assigns a high feature importance to WT and a low importance to FFM, because the contribution of FFM is no longer needed after inclusion of WT. In the PM model, on the other hand, FFM was selected as more important than WT. This might be due to the fact that PM and ML are two very different methods, which select features in a different way. However, this should not have a large impact on the results since FFM and WT are directly correlated and assigning one a high and the other one a low importance would likely describe the data very similarly. In PM, the true covariate FFM would be added as a covariate on, e.g., drug clearance or drug volume of distribution, indicating which process the covariate influences. This information, however, is not easily obtainable from ML models, and it thus remains unknown how exactly the exposure is influenced by a feature. Another difference is that in PM TAD, OCC and dose are a part of the structural model, and are always used for prediction of concentration over time in population PK modelling, while in ML these are considered features, just like FFM.

Using ML instead of PM could be promising for prediction of PK due to its time- and labor-efficiency in situations when the gold standard population PK cannot be used, or there is no need for a more mechanistic understanding of the PK, or to develop a model that can perform simulations. All the tested ML algorithms in this study outperformed the PM model with respect to run time, being at least 22 times faster. Considering the fact that PM model development is a manual, stepwise procedure, it is also important to note that a PM model has to be run repeatedly, especially when performing covariate analysis. It is often necessary to run the model 20–100 times until the final model is reached. In ML, however, many algorithms have an embedded feature selection, i.e., the model only has to be run once. A fairer comparison would thus be a multiple of 11,479 s (191 min) for the PM model, depending on how many developmental steps are involved and how many covariates are explored, versus one run time for the ML model plus the run time for hyperparameter tuning. However, both pure run times and the model development itself are faster using ML. Due to the stepwise, manual model building process in PM, it usually takes several weeks for an experienced modeller to develop a population PK model, while an ML model can most often be developed in less time. With large amounts of diverse data, the time ratio would change even more. This increased efficiency is especially beneficial in light of the good predictive performance of the nonlinear ML models explored in this study when at least two plasma concentrations are used as input. In this work, we demonstrated a good precision and accuracy in the ML predictions for both longitudinal data (XGBoost: RMSE: 6.9 mg/L, MAE: 4.0 mg/L) and exposure indices (AUC_0–24h_) (LASSO: RMSE: 29.1 h·mg/L, MAE: 18.8 h·mg/L), using six concentrations as input. In both cases, the inclusion of observed rifampicin plasma concentrations as features considerably improved the model performance.

For prediction of the plasma concentration-time series, the predictions (Figure 5) indicated that the nonlinear ML algorithms, especially XGBoost, were in good accordance with the data simulated from the population PK model, and the shape of the concentration-time profiles was predicted accurately (Figure 6 and Figure 7). Despite the high complexity in rifampicin PK, including autoinduction of elimination, concentration-dependent nonlinear clearance, dose-dependent bioavailability [14,15,16] and high IOV [17], the nonlinear ML models performed well when at least two plasma concentrations were used as input. The results showed that the best-performing model (XGBoost) was able to predict accurately at treatment weeks 1 and 2 (Figure 7), suggesting that the model can handle autoinduction [14,15]. The good performance across all dose groups (Figure 7) indicated that dose-dependent bioavailability and concentration-dependent elimination are handled properly. However, the XGBoost predictions may have a weakness in identifying the true upper range of high exposure (see Figure 6). When comparing the individual predicted plasma concentration-time profiles to the data simulated from the population PK model, here considered to be observed data, it becomes obvious that there is a larger variability in the observed data (95% PI predictions: 62.6 mg/L, 95% PI observations: 69.4 mg/L) (see Figure 6 and Figure 8). This could be due to the fact that the population PK model incorporates different sources of variability, such as IIV, IOV and residual error, and is thus able to describe variability between patients and within a patient well. The ML algorithms explored in this study, however, only use the features as a source of variability and cannot account for IIV that is not caused by any of the features. This could be important to appreciating/predicting risks of individual patients reaching a safety threshold of exposure.

For prediction of AUC_0–24h_, all four algorithms performed well when at least two plasma concentrations were used as input. The results clearly show a correlation between the amount of data used for training and model performance. Using features only without rifampicin plasma concentrations as input, resulted in weak model performance with an *R*^2^ of 0.41, RMSE of 117.9 h·mg/L and MAE of 74.2 h·mg/L (LASSO). Using two plasma concentrations at 2 h and 4 h post-dose as input, resembling a limited sampling strategy [64], led to a higher model performance (LASSO, *R*^2^: 0.69, RMSE: 86.8 h·mg/L, MAE: 54.5 h·mg/L). The best predictive performance was achieved when using six plasma concentrations as input, representing a richer sampling, where LASSO performed very well with an *R*^2^ of 0.97 (RMSE: 29.1 h·mg/L, MAE: 18.8 h·mg/L) (see also Table 4). This indicates that predicting AUC_0–24h_ accurately and precisely without drug plasma concentrations is challenging. At least two concentrations are needed to reach acceptable model performance.

The generalizability of the algorithms to predict well across all test sets is important to evaluate overfitting and identify outliers. The generalizability of the models across the test sets obtained from the five-fold cross-validation was acceptable as the range of RMSE and MAE was in general spread evenly around the average value (see Table 3 and Table 4), with the exception of GBM in scenario 6.

Due to the different nature of the evaluated algorithms (linear versus nonlinear), they performed very differently for prediction of longitudinal data and AUC_0–24h_. While the linear model LASSO showed excellent performance for AUC_0–24h_ prediction using six concentrations (*R*^2^: 0.97), it was not able to predict longitudinal data (*R*^2^: 0.39). This is likely due to the different nature of the longitudinal data and the AUC_0–24h_. A concentration-time series has a distinct shape (see, e.g., Figure 6), which a linear algorithm such as LASSO is not able to describe. The AUC_0–24h_, however, is a summary variable, describing the whole time-series in one value. This simplifies the problem and enables even a linear algorithm to predict well. The nonlinear models performed well for both prediction of longitudinal data as well as AUC_0–24h_, including at least two plasma concentrations as input. However, the PM model using NLME methodology still performs better for prediction of longitudinal data, as shown in Figure 8. NLME models are better able to capture the variability between and within patients compared to the ML models investigated here (Figure 6 and Figure 8). There is thus a need for further studies investigating how the variability could be better captured using ML.

PK data are naturally imbalanced due to the fact that plasma PK sampling is often performed very sparsely. This can often lead to poor performance in areas of sparse information, e.g., around the C_max_ using ML. One approach addressing this issue is oversampling, which is a method increasing the data size in sparse areas. Due to the fact that PK data is not assumed to be normally distributed, we did not apply oversampling in this work, but believe that oversampling methods appropriate for sparse PK data should be investigated.

In this work, we used an exemplary simplified case study and time efficiencies provided an example of a proof of value study. ML is a purely data-driven approach and should thus be regarded as an assistive tool rather than a decision maker at this stage. Richer, more diverse data will help produce a more accurate tool, but in this study, we exemplified the potential of ML. In this study, the model performance between PM and ML could not be directly compared, since the data were simulated from the PM model, which might bias PM model performance. There is thus a need for further studies comparing the performance of PM and ML directly using real patient data. In addition, the generalizability of the ML methods should be investigated further. A limitation of this work is that data simulated from a population PK model instead of real patient data were used as input for the ML model training. Even though real patient data would have reflected the real-world scenario perfectly, we believe that using simulated data instead did not impact the results significantly, since the population PK model used to perform the simulations [15] has been validated externally and shown to predict real patient data accurately and precisely [64]. Moreover, in order to be able to make the simulated dataset in Supplementary Materials Data S1 public, the patient covariates were sampled from a parametric covariate distribution of the HIGHRIF1 trial [65] and not bootstrapped. Even though bootstrap is the gold standard method, it is assumed that the results do not vary much, since the correlations between covariates were retained and the sampling was performed from a truncated distribution using the reported ranges as minimum and maximum values. One drawback of the ML model is that some plasma concentrations were predicted to be negative, which is not the case in the simulated data, as the lowest possible prediction is restricted to 0 mg/L due to scientific plausibility. Restricting the ML models or working with log-transformed data for future work could be one way to avoid this issue. In addition, we see a need to do more work on inflation of classifier accuracy due to data crosstalk. Furthermore, the time-points resembling sparse PK sampling (2 and 4 h post-dose) are based on a PM study [64]. A sparse sampling strategy in PM is often based on gaining information on exposure during the absorption and elimination phase. This might not be the case for ML models, and thus there is a need to investigate an optimal sampling strategy appropriate for ML.

There are few studies investigating how ML and PM could be combined to further improve drug development [5,8,9,10,11,12]. This work is one of the most comprehensive illustrative examples of a side-by-side comparison of the two methods. We here exemplify in a case study the use of ML for PK predictions, which could then be used as input into a PKPD model, enabling faster but still accurate PKPD analysis. We demonstrate that ML could be a useful tool for PK analysis, providing fast predictions of both the full concentration time series as well as PK exposure indices (e.g., AUC_0–24h_) with acceptable precision. Further work is needed to investigate this tool using real patient data. Bridging the gap between PM and ML seems promising considering that ML can add value to PM workflows through increased time and labor efficiency.

## Figures and Tables

**Table 2:** Different scenarios of data sizes used for model training and predicted outcome.

Scenario	Model	Predictions
1	Features only	Rifampicin concentration-time series ^c^
2	Features + 2 observed rifampicin concentrations ^a^	Rifampicin concentration-time series ^c^
3	Features + 6 observed rifampicin concentrations ^b^	Rifampicin concentration-time series ^c^
4	Features only	AUC_0–24h_
5	Features + 2 observed rifampicin concentrations ^a^	AUC_0–24h_
6	Features + 6 observed rifampicin concentrations ^b^	AUC_0–24h_

^a^ Time-points of rifampicin concentrations are at 2 and 4 h post-dose at days 7 and 14, representing a sparse sampling schedule. ^b^ Time-points of rifampicin concentrations are at 0.5, 1, 2, 4, 8 and 24 h post-dose at days 7 and 14, representing a richer sampling schedule. ^c^ At pre-dose and 0.5, 1, 1.5, 2, 3, 4, 6, 8, 12, and 24 h post-dose at days 7 and 14. AUC_0–24h_, area under the rifampicin plasma concentration-time curve up to 24 h.

**Table 3:** Model performance for prediction of plasma concentration over time using varying amounts of information as input.

	GBM			XGBoost			Random Forest			LASSO		
	Scenario 1	Scenario 2	Scenario 3	Scenario 1	Scenario 2	Scenario 3	Scenario 1	Scenario 2	Scenario 3	Scenario 1	Scenario 2	Scenario 3
*R* ^2^	0.57	0.76	0.83	0.60	0.76	0.84	0.54	0.75	0.82	0.25	0.36	0.39
Pearson correlation	0.77	0.87	0.90	0.78	0.87	0.91	0.75	0.86	0.90	0.52	0.62	0.65
RMSE (mg/L)	10.9 (8.9–13.3)	8.3 (6.8–8.6)	7.1 (5.1–7.3)	10.6 (8.9–13.5)	8.3 (6.7–12.4)	6.9 (5.1–11.1)	11.3 (9.8–14.1)	8.5 (6.9–12.7)	7.2 (5.3–11.8)	14.5 (13.4–19.1)	13.3 (11.5–16.6)	12.9 (11.3–15.3)
MAE (mg/L)	7.1 (6.0–7.1)	5.2 (4.3–6.8)	4.1 (3.3–5.7)	7.0 (6.0–8.0)	5.1 (4.2–6.7)	4.0 (3.2–5.4)	7.0 (6.4–8.0)	4.9 (4.2–6.4)	3.8 (2.8–5.3)	10.2 (9.9–12.2)	9.6 (8.4–11.1)	9.3 (8.1–10.5)
Run time (s)	6.8	8.2	11.1	1.4	1.2	4.7	309.9	362.6	508.7	1.1	1.3	1.1

In scenario 1, the models were trained to predict the rifampicin plasma concentration-time series (11 time-points at days 7 and 14) based only on features (no plasma concentrations). In scenario 2, the models were trained to predict the rifampicin plasma concentration-time series (11 time-points at days 7 and 14) based on features and 2 plasma concentrations at time-points 2 and 4 h post-dose at days 7 and 14. In scenario 3, the models were trained to predict the rifampicin plasma concentration-time series (11 time-points at days 7 and 14) based on features and 6 plasma concentrations at time-points 0.5, 1, 2, 4, 8 and 24 h post-dose at days 7 and 14. MAE, mean absolute error averaged across the n-folds (range); RMSE, root mean square error averaged across the n-folds (range).

**Table 4:** Model performance for prediction of rifampicin AUC_0-24h_ using varying amounts of information as input.

	GBM			XGBoost			Random Forest			LASSO		
	Scenario 4	Scenario 5	Scenario 6	Scenario 4	Scenario 5	Scenario 6	Scenario 4	Scenario 5	Scenario 6	Scenario 4	Scenario 5	Scenario 6
*R* ^2^	0.27	0.61	0.73	0.44	0.71	0.84	0.22	0.62	0.78	0.41	0.69	0.97
Pearson correlation	0.59	0.73	0.83	0.63	0.75	0.83	0.55	0.73	0.83	0.67	0.84	0.98
RMSE (h·mg/L)	131.7 (86.9–246.6)	103.0 (49.8–233.1)	88.2 (41.7–218.2)	121.0 (57.7–262.8)	92.6 (38.9–250.1)	69.6 (21.0–238.3)	137.1 (76.8–252.8)	103.5 (48.5–238.7)	79.9 (30.2–208.5)	117.9 (76.0–238.5)	86.8 (48.3–175.5)	29.1 (20.7–57.3)
MAE (h·mg/L)	85.5 (74.4–121.1)	61.3 (43.2–105.8)	47.6 (21.0–238.3)	76.7 (47.1–122.3)	52.6 (30.5–110.5)	30.4 (13.3–82.8)	84.6 (63.1–118.3)	59.4 (39.6–102.6)	38.3 (22.5–74.8)	74.2 (56.5–119.7)	54.5 (38.4–87.2)	18.8 (15.2–29.2)
Run time (s)	1.3	1.6	1.8	0.7	4.7	4.1	20.5	21.9	22.8	1.1	1.0	1.1

In scenario 4, the models were trained to predict rifampicin AUC_0–24h_ based only on features (no plasma concentrations) at days 7 and 14. In scenario 5, the models were trained to predict rifampicin AUC_0–24h_ based on features and 2 plasma concentrations at time-points 2 and 4 h post-dose at days 7 and 14. In scenario 6, the models were trained to predict rifampicin AUC_0–24h_ based on features and 6 plasma concentrations at time-points 0.5, 1, 2, 4, 8 and 24 h post-dose at days 7 and 14. AUC_0–24h_, Area under the rifampicin plasma concentration-time curve from 0 to 24 h; MAE, mean absolute error averaged across the n-folds (range); RMSE, root mean square error averaged across the n-folds (range).

## Data Availability

The simulated dataset can be found in Supplementary Materials Data S1.

## Supplementary Materials

The following supporting information can be downloaded at: https://www.mdpi.com/article/10.3390/pharmaceutics14081530/s1, Data S1: Simulated dataset, Text S2: Final model code. Figure S1. Illustration of the different types of variability in a nonlinear mixed effects model. The plasma concentration time-curve for a fictive drug is shown. The blue line represents the model predictions for the typical individual and the orange line the model predictions for one specific individual. The blue dots represent the observations of all individuals in the population and the orange dots the observations for the one specific individual. The covariates for the blue and orange lines are consistent. The difference between typical pharmacokinetic (PK) parameters (population PK parameters) and the individual PK parameters is expressed as inter-individual variability (IIV), and is reflected by the difference in drug exposure between the typical individual (blue line) and an individual (orange line). The remaining difference between individual model predictions (orange line) and observed concentrations (orange dots) are described by residual unexplained variability (RUV). Figure S2: Importance scores for evaluated features shown for the machine learning algorithms GBM, Random forest, XGBoost and LASSO for (A) AUC_0–24h_ predictions using features only as input (scenario 4), (B) AUC_0–24h_ predictions using 2 plasma concentrations as input (scenario 5), (C) AUC_0–24h_ predictions using 6 plasma concentrations as input (scenario 6), (D) prediction of the plasma concentration-time series using 2 plasma concentrations as input (scenario 2), (E) prediction of the plasma concentration-time series using 6 plasma concentrations as input (scenario 3). AGE, age (years); BMI, body mass index (kg/m^2^); DOSE, daily rifampicin dose (mg); FFM, fat-free mass (kg); HIV, HIV-coinfection; HT, body height (cm); OCC, treatment week, RACE, race; SEX, gender; TAD, time after dose (h); WT, bodyweight (kg); TAD, time after dose (h); TAD_0.5, rifampicin plasma concentration at 0.5 h post-dose; TAD_1, rifampicin plasma concentration at 1 h post-dose; TAD_2, rifampicin plasma concentration at 2 h post-dose; TAD_4, rifampicin plasma concentration at 4 h post-dose; TAD_8, rifampicin plasma concentration at 8 h post-dose; TAD_24, rifampicin plasma concentration at 24 h post-dose. Figure S3: Individual rifampicin plasma concentrations predicted from the eXtreme Gradient Boosting (XGBoost) model (solid line and open circles) compared to the true concentrations (black closed circles). Panel (A) represents the predictions for each individual in the test dataset at the first week of rifampicin treatment for scenario 1 (predictions based on features only). Panel (B) represents the predictions for each individual in the test dataset at the second week of rifampicin treatment for scenario 1 (predictions based on features only). Panel (C) represents the predictions for each individual in the test dataset at the first week of rifampicin treatment for scenario 2 (predictions based on features and 2 rifampicin plasma concentrations). Panel (D) represents the predictions for each individual in the test dataset at the second week of rifampicin treatment for scenario 2 (predictions based on features and 2 rifampicin plasma concentrations). The different colors indicate the different daily rifampicin doses. Table S1. Final model hyperparameters for the different machine learning models.
