# Supplementary material for: Machine Learning and Pharmacometrics for Prediction of Pharmacokinetic Data: Differences, Similarities and Challenges Illustrated with Rifampicin
Source: Pharmaceutics. 2022 Jul 22;14(8):1530. doi: 10.3390/pharmaceutics14081530 (PMC9330804; doi:10.3390/pharmaceutics14081530)
Supplement: Supplementary file 1 [file pharmaceutics-14-01530-s001.zip › Supplementary Figure S1.pdf]

Supplementary Figure S1

Supplement to:

Title: Machine learning and pharmacometrics for prediction of pharmacokinetic data: Differences, similarities and challenges illustrated with rifampicin

Authors: Lina Keutzer[1¥], Huifang You[1¥], Ali Farnoud[2], Joakim Nyberg[3], Sebastian G Wicha[4], Gareth Maher-Edwards[5], Georgios Vlasakakis[5], Gita Khalili Moghaddam[5], Elin M Svensson[3,8], Michael Menden[3,6,7], Ulrika SH Simonsson[1*]

Affiliations:

[1]Department of Pharmaceutical Biosciences, Uppsala University, Uppsala, Sweden, Ulrika.Simonsson@farmbio.uu.se;
[2]Institute of Computational Biology, Helmholtz Zentrum München, Munich, Germany;
[3]Department of Pharmacy, Uppsala University, Uppsala, Sweden;
[4]Department of Clinical Pharmacy, Institute of Pharmacy, University of Hamburg, Hamburg, Germany;
[5]Research, Clinical Pharmacology Modelling & Simulation, GlaxoSmithKline, London, UK;
[6]Department of Biology, Ludwig-Maximilians University Munich, Martinsried, Germany;
[7]German Center for Diabetes Research (DZD e.V.), Neuherberg, Germany;
[8] Department of Pharmacy, Radboud institute of health sciences, Radboud university medical center, Nijmegen, The Netherlands

[¥] These authors contributed equally

*Correspondence: Ulrika.Simonsson@farmbio.uu.se, Department of Pharmaceutical Biosciences, Uppsala University, Box 591, 75124 Uppsala, Sweden;

Supplementary Figure S1

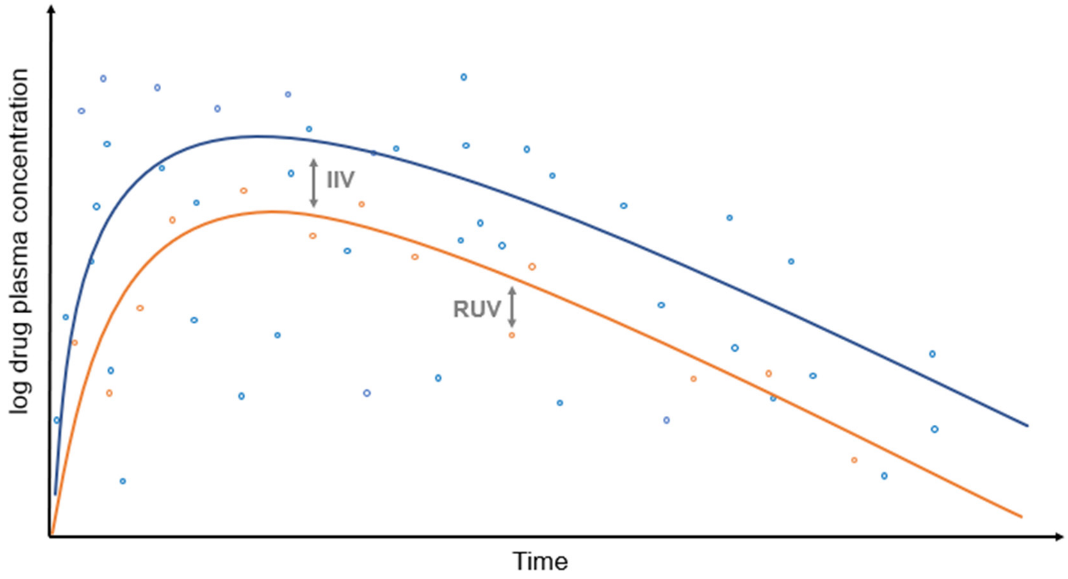

Figure S1. Illustration of the different types of variability in a nonlinear mixed effects model. The plasma concentration time-curve for a fictive drug is shown. The blue line represents the model predictions for the typical individual and the orange line the model predictions for one specific individual. The blue dots represent the observations of all individuals in the population and the orange dots the observations for the one specific individual. The covariates for the blue and orange lines are consistent. The difference between typical pharmacokinetic (PK) parameters (population PK parameters) and the individual PK parameters is expressed as inter-individual variability (IIV), and is reflected by the difference in drug exposure between the typical individual (blue line) and an individual (orange line). The remaining difference between individual model predictions (orange line) and observed concentrations (orange dots) are described by residual unexplained variability (RUV).
