# Supplementary material for: Machine Learning and Pharmacometrics for Prediction of Pharmacokinetic Data: Differences, Similarities and Challenges Illustrated with Rifampicin
Source: Pharmaceutics. 2022 Jul 22;14(8):1530. doi: 10.3390/pharmaceutics14081530 (PMC9330804; doi:10.3390/pharmaceutics14081530)
Supplement: Supplementary file 1 [file pharmaceutics-14-01530-s001.zip › Supplementary Figure S2.pdf]

Supplementary Figure S2

Supplement to:

Title: Machine learning and pharmacometrics for prediction of pharmacokinetic data: Differences, similarities and challenges illustrated with rifampicin

Authors: Lina Keutzer[1¥], Huifang You[1¥], Ali Farnoud[2], Joakim Nyberg[3], Sebastian G Wicha[4], Gareth Maher-Edwards[5], Georgios Vlasakakis[5], Gita Khalili Moghaddam[5], Elin M Svensson[3,8], Michael Menden[3,6,7], Ulrika SH Simonsson[1*]

Affiliations:

[1]Department of Pharmaceutical Biosciences, Uppsala University, Uppsala, Sweden, Ulrika.Simonsson@farmbio.uu.se;
[2]Institute of Computational Biology, Helmholtz Zentrum München, Munich, Germany;
[3]Department of Pharmacy, Uppsala University, Uppsala, Sweden;
[4]Department of Clinical Pharmacy, Institute of Pharmacy, University of Hamburg, Hamburg, Germany;
[5]Research, Clinical Pharmacology Modelling & Simulation, GlaxoSmithKline, London, UK;
[6]Department of Biology, Ludwig-Maximilians University Munich, Martinsried, Germany;
[7]German Center for Diabetes Research (DZD e.V.), Neuherberg, Germany;
[8] Department of Pharmacy, Radboud institute of health sciences, Radboud university medical center, Nijmegen, The Netherlands

[¥] These authors contributed equally

*Correspondence: Ulrika.Simonsson@farmbio.uu.se, Department of Pharmaceutical Biosciences, Uppsala University, Box 591, 75124 Uppsala, Sweden;

Supplementary Figure S2

A)

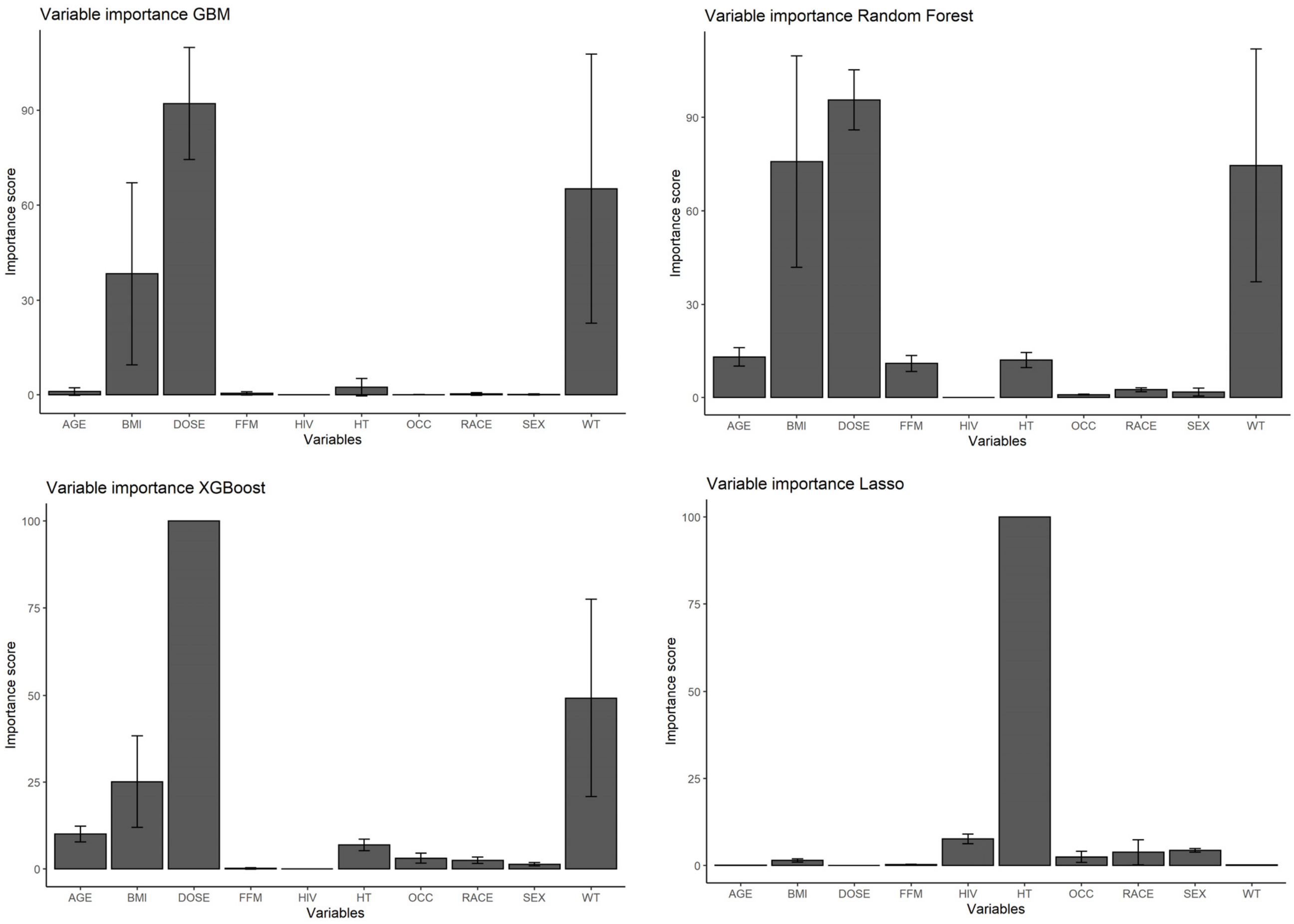

B)

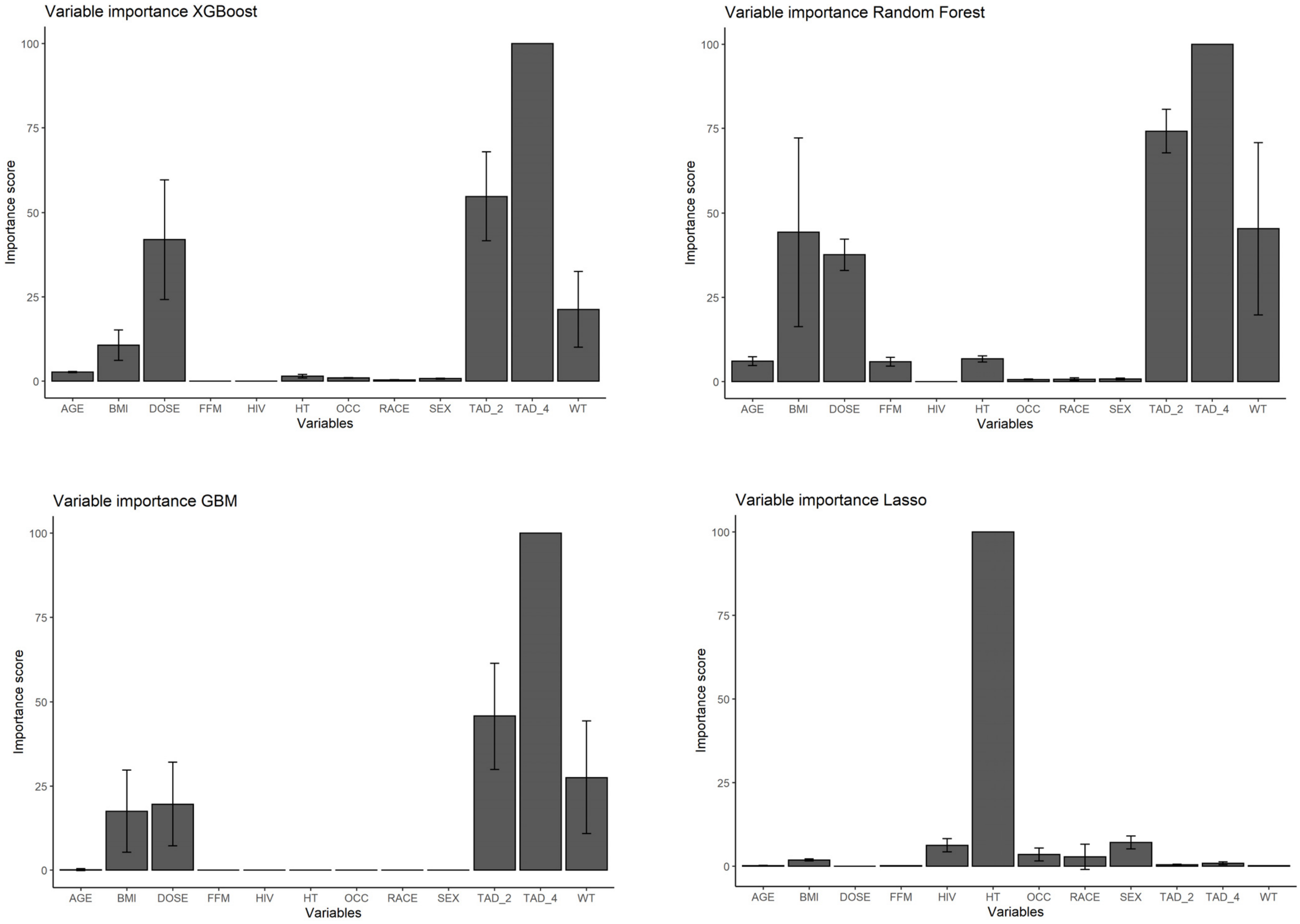

Variable importance XGBoost
Importance score
100
75
50
25
0
AGE
BMI
DOSE
FFM
HIV
HT
OCC
RACE
SEX
TAD_2
TAD_4
WT
Variables
Variable importance Random Forest
Importance score
Variables
Variable importance GBM
Importance score
Variables
Variable importance Lasso
Importance score
Variables

C)

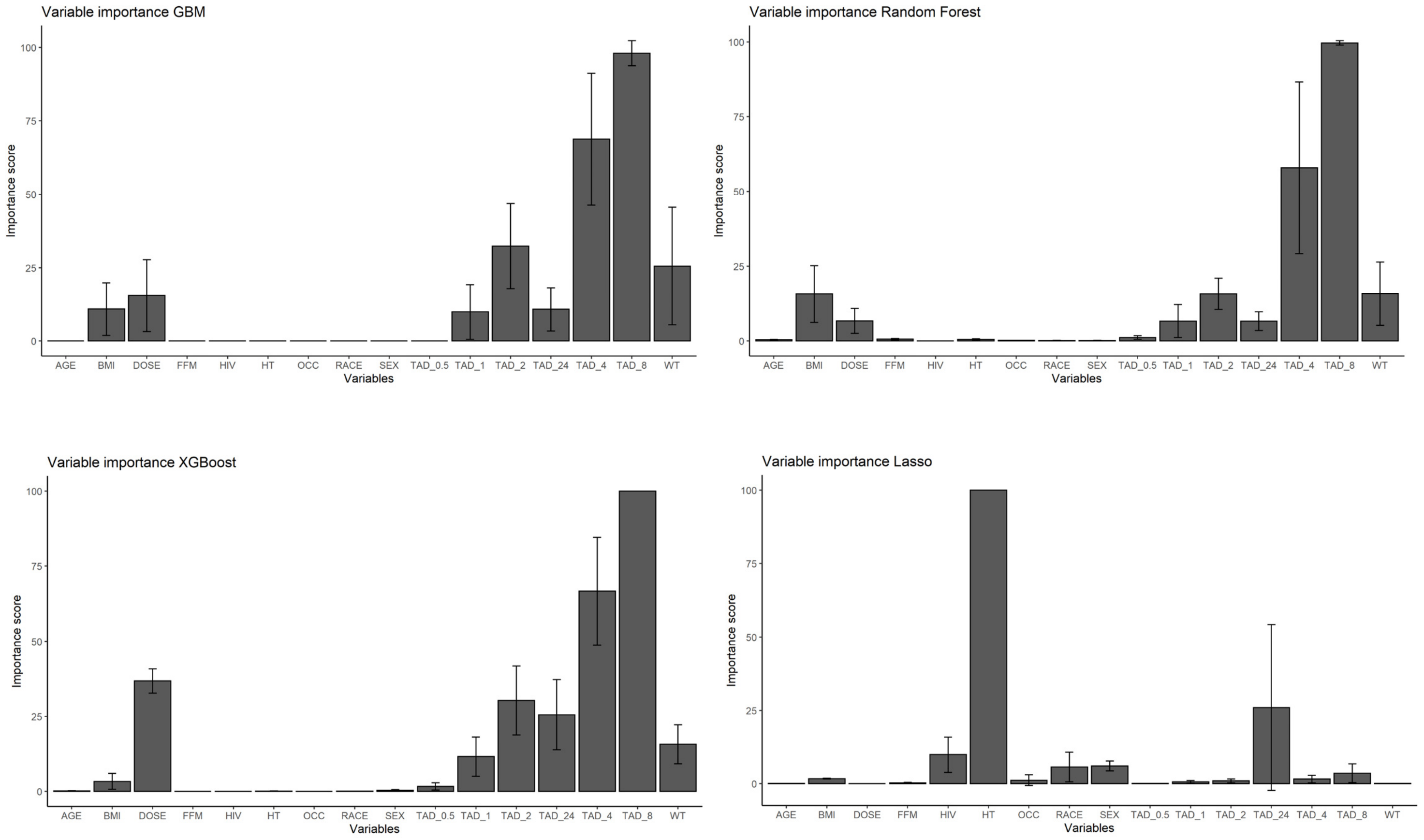
Variable importance GBM
Variable importance Random Forest
Variable importance XGBoost
Variable importance Lasso
Importance score
Variables
0
25
50
75
100
AGE
BMI
DOSE
FFM
HIV
HT
OCC
RACE
SEX
TAD_0.5
TAD_1
TAD_2
TAD_24
TAD_4
TAD_8
WT

D)

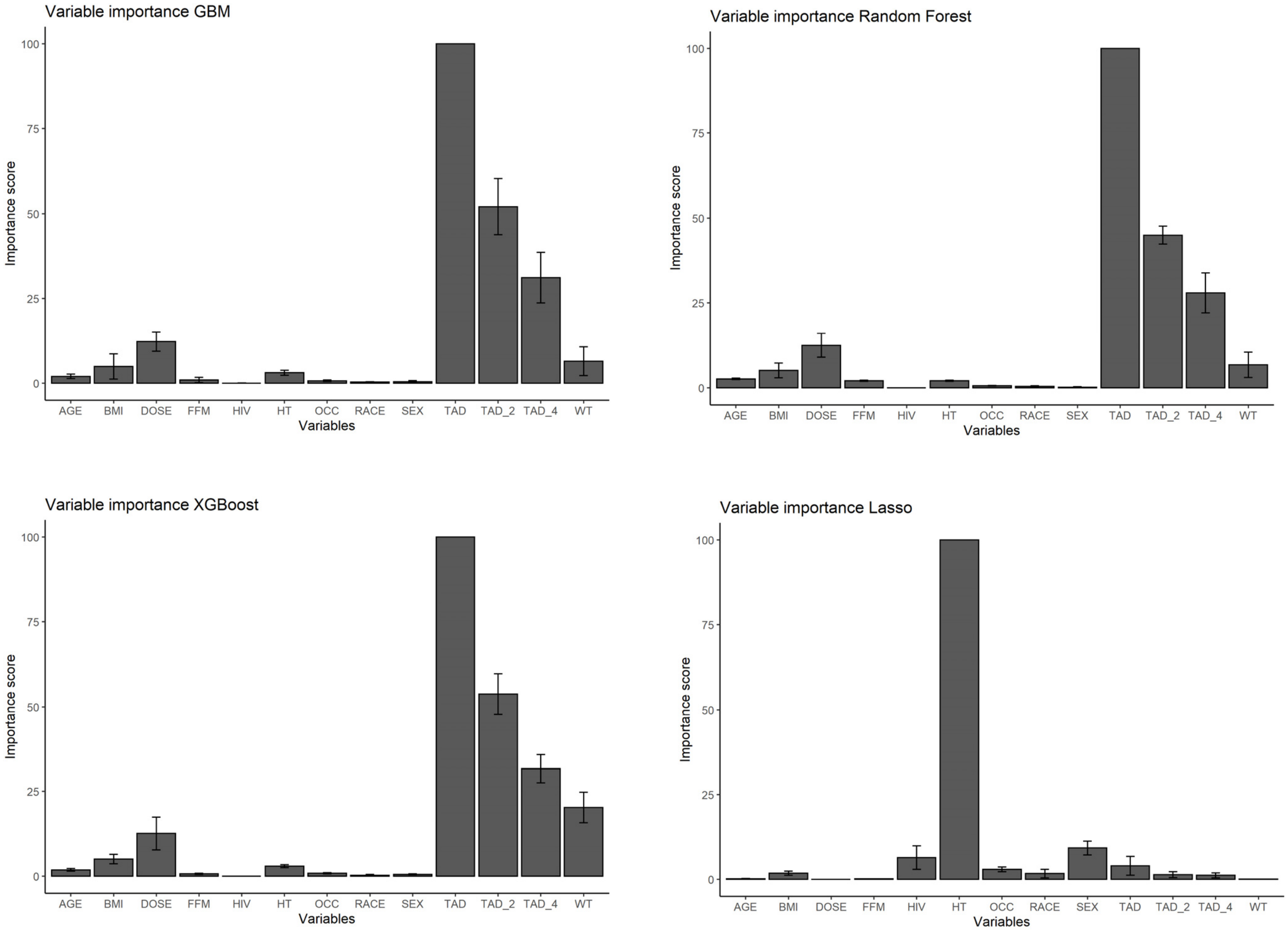
Variable importance GBM
Variable importance Random Forest
Variable importance XGBoost
Variable importance Lasso
Importance score
Variables
0
25
50
75
100
AGE
BMI
DOSE
FFM
HIV
HT
OCC
RACE
SEX
TAD
TAD_2
TAD_4
WT

E)

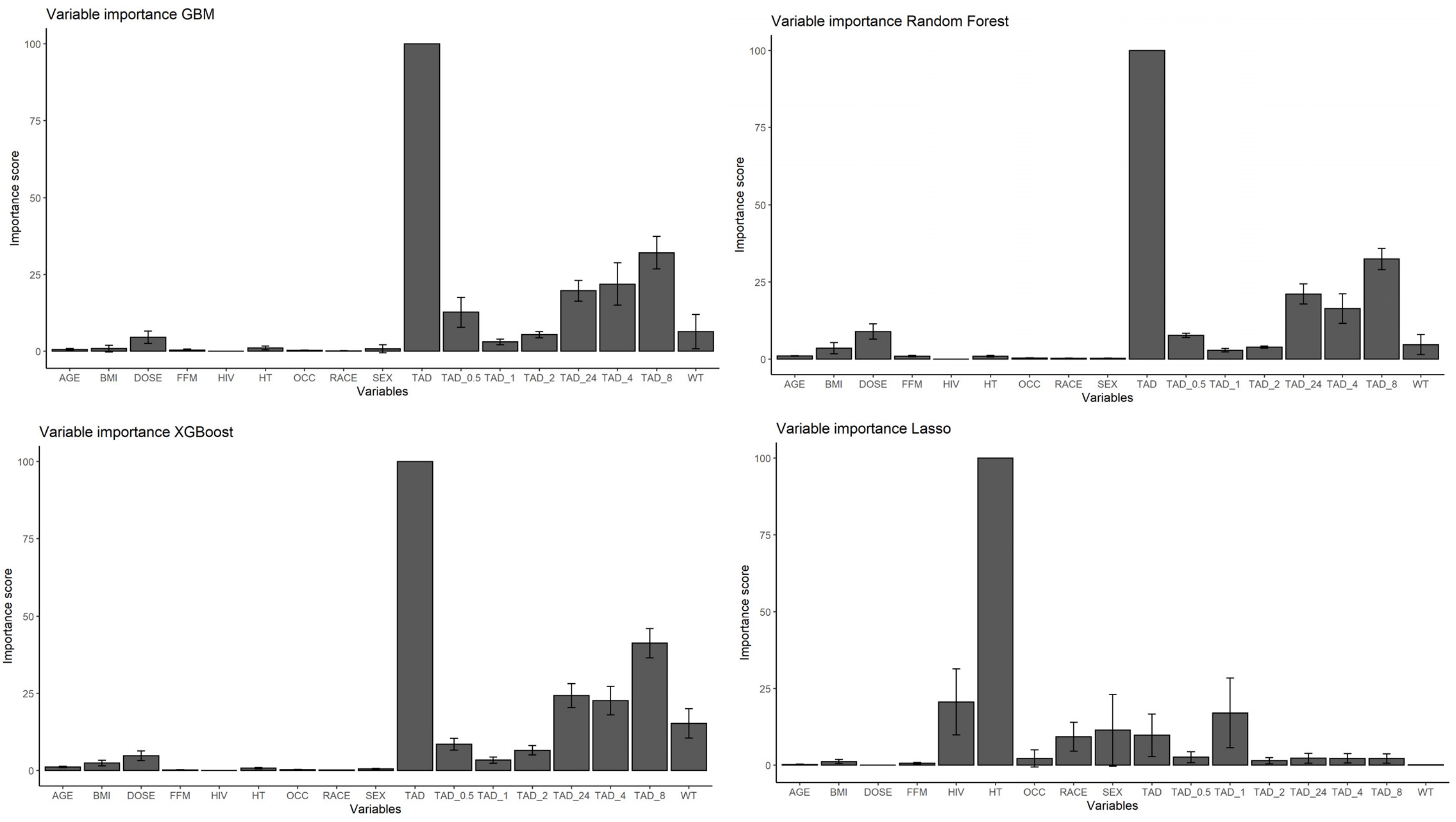

Figure S2. Importance scores for evaluated features shown for the machine learning algorithms GBM, Random forest, XGBoost and LASSO for A) $AUC_{0\text{-}24h}$ predictions using features only as input (scenario 4), B) $AUC_{0\text{-}24h}$ predictions using 2 plasma concentrations as input (scenario 5), C) $AUC_{0\text{-}24h}$ predictions using 6 plasma concentrations as input (scenario 6), D) prediction of the plasma concentration-time series using 2 plasma concentrations as input (scenario 2), E) prediction of the plasma concentration-time series using 6 plasma concentrations as input (scenario 3). AGE, age (years); BMI, body mass index (kg/m2); DOSE, daily rifampicin dose (mg); FFM, fat-free mass (kg); HIV, HIV-coinfection; HT, body height (cm); OCC, treatment week, RACE, race; SEX, gender; TAD, time after dose (h); WT, bodyweight (kg); TAD, time after dose (h); TAD_0.5, rifampicin plasma concentration at 0.5 hours post-dose; TAD_1, rifampicin plasma concentration at 1 hours post-dose; TAD_2, rifampicin plasma concentration at 2 hours post-dose; TAD_4, rifampicin plasma concentration at 4 hours post-dose; TAD_8, rifampicin plasma concentration at 8 hours post-dose; TAD_24, rifampicin plasma concentration at 24 hours post-dose.
