# Supplementary material for: Machine Learning and Pharmacometrics for Prediction of Pharmacokinetic Data: Differences, Similarities and Challenges Illustrated with Rifampicin
Source: Pharmaceutics. 2022 Jul 22;14(8):1530. doi: 10.3390/pharmaceutics14081530 (PMC9330804; doi:10.3390/pharmaceutics14081530)
Supplement: Supplementary file 1 [file pharmaceutics-14-01530-s001.zip › Supplementary Figure S3.pdf]

Supplementary Figure S3

Supplement to:

Title: Machine learning and pharmacometrics for prediction of pharmacokinetic data: Differences, similarities and challenges illustrated with rifampicin

Authors: Lina Keutzer[1¥], Huifang You[1¥], Ali Farnoud[2], Joakim Nyberg[3], Sebastian G Wicha[4], Gareth Maher-Edwards[5], Georgios Vlasakakis[5], Gita Khalili Moghaddam[5], Elin M Svensson[3,8], Michael Menden[3,6,7], Ulrika SH Simonsson[1*]

Affiliations:

[1]Department of Pharmaceutical Biosciences, Uppsala University, Uppsala, Sweden, Ulrika.Simonsson@farmbio.uu.se;

[2]Institute of Computational Biology, Helmholtz Zentrum München, Munich, Germany;

[3]Department of Pharmacy, Uppsala University, Uppsala, Sweden;

[4]Department of Clinical Pharmacy, Institute of Pharmacy, University of Hamburg, Hamburg, Germany;

[5]Research, Clinical Pharmacology Modelling & Simulation, GlaxoSmithKline, London, UK;

[6]Department of Biology, Ludwig-Maximilians University Munich, Martinsried, Germany;

[7]German Center for Diabetes Research (DZD e.V.), Neuherberg, Germany;

[8] Department of Pharmacy, Radboud institute of health sciences, Radboud university medical center, Nijmegen, The Netherlands

[¥] These authors contributed equally

*Correspondence: Ulrika.Simonsson@farmbio.uu.se, Department of Pharmaceutical Biosciences, Uppsala University, Box 591, 75124 Uppsala, Sweden;

Supplementary Figure S3

A)

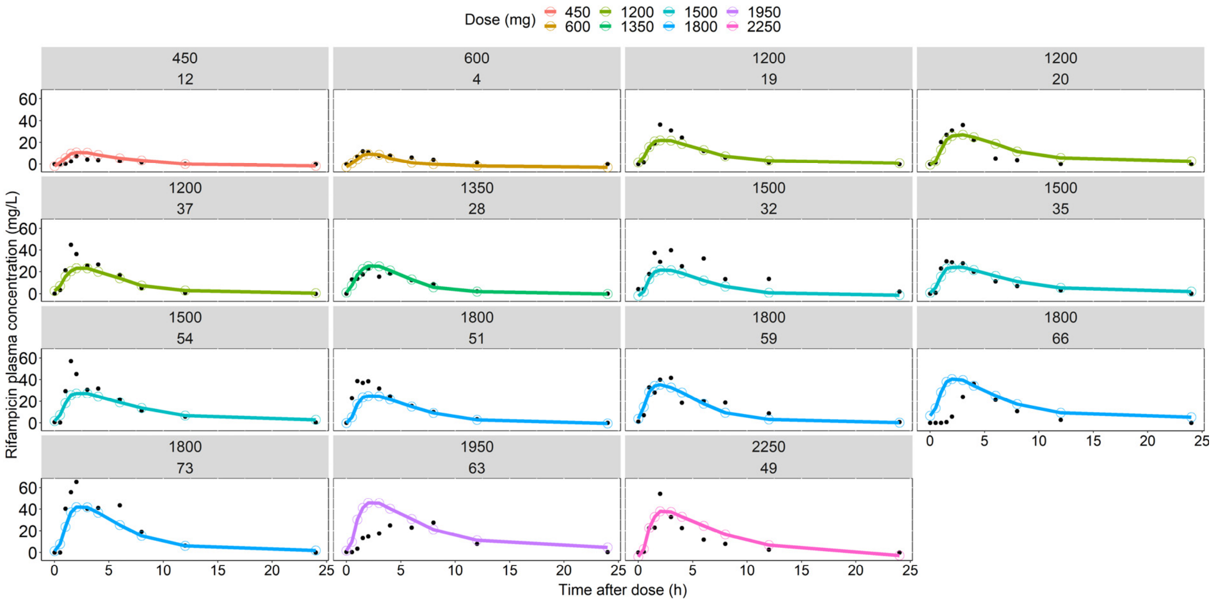

B)

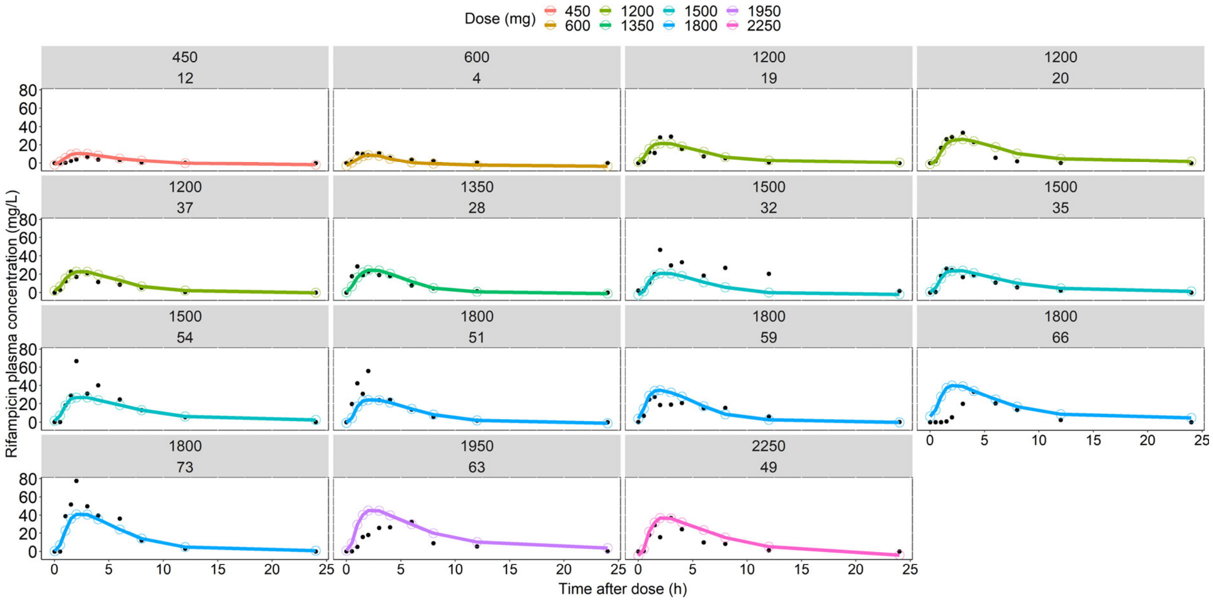

C)

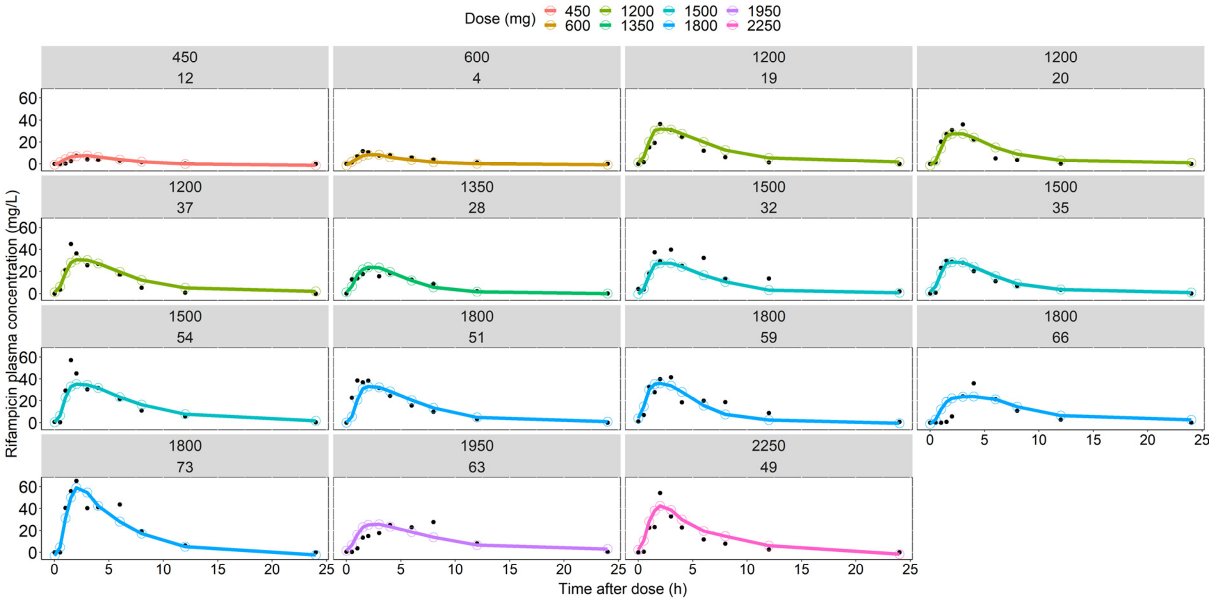

D)

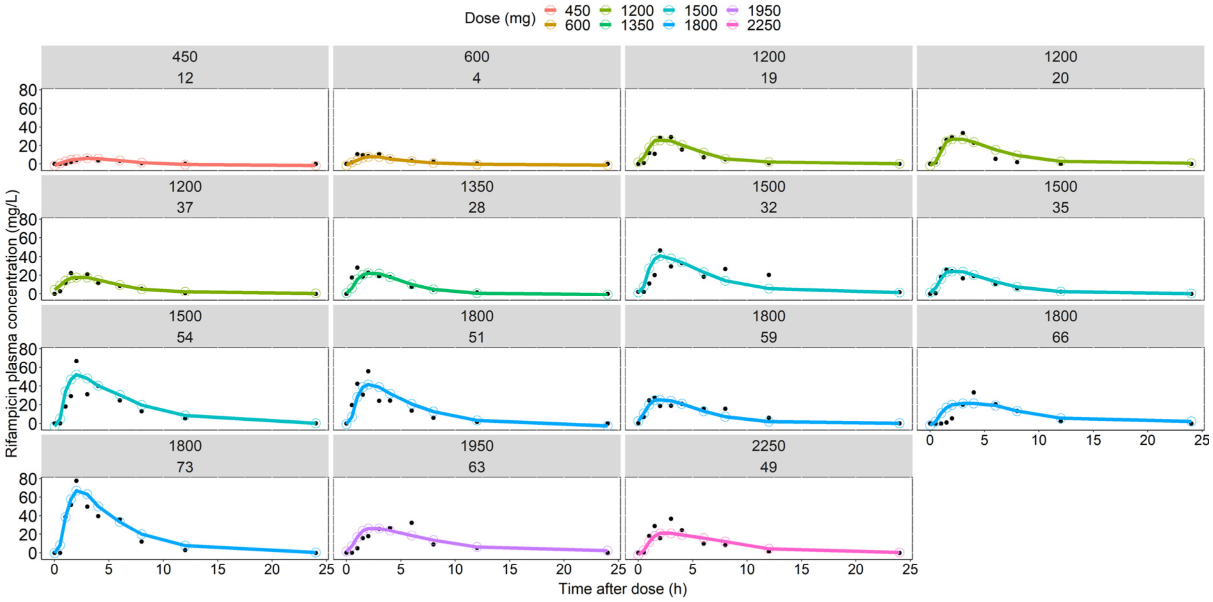

Figure S3. Individual rifampicin plasma concentrations predicted from the eXtreme Gradient Boosting (XGBoost) model (solid line and open circles) compared to the true concentrations (black closed circles). Panel A) represents the predictions for each individual in the test dataset at the first week of rifampicin treatment for scenario 1 (predictions based on features only). Panel B) represents the predictions for each individual in the test dataset at the second week of rifampicin treatment for scenario 1 (predictions based on features only). Panel C) represents the predictions for each individual in the test dataset at the first week of rifampicin treatment for scenario 2 (predictions based on features and 2 rifampicin plasma concentrations). Panel D) represents the predictions for each individual in the test dataset at the second week of rifampicin treatment for scenario 2 (predictions based on features and 2 rifampicin plasma concentrations). The different colors indicate the different daily rifampicin doses.
