# Supplementary material for: Machine Learning and Pharmacometrics for Prediction of Pharmacokinetic Data: Differences, Similarities and Challenges Illustrated with Rifampicin
Source: Pharmaceutics. 2022 Jul 22;14(8):1530. doi: 10.3390/pharmaceutics14081530 (PMC9330804; doi:10.3390/pharmaceutics14081530)
Supplement: Supplementary file 1 [file pharmaceutics-14-01530-s001.zip › Supplementary Table S1.pdf]

Supplementary Table S1

Supplement to:

Title: Machine learning and pharmacometrics for prediction of pharmacokinetic data: Differences, similarities and challenges illustrated with rifampicin

Authors: Lina Keutzer[1¥], Huifang You[1¥], Ali Farnoud[2], Joakim Nyberg[3], Sebastian G Wicha[4], Gareth Maher-Edwards[5], Georgios Vlasakakis[5], Gita Khalili Moghaddam[5], Elin M Svensson[3,8], Michael Menden[3,6,7], Ulrika SH Simonsson[1*]

Affiliations:

[1]Department of Pharmaceutical Biosciences, Uppsala University, Uppsala, Sweden, Ulrika.Simonsson@farmbio.uu.se;
[2]Institute of Computational Biology, Helmholtz Zentrum München, Munich, Germany;
[3]Department of Pharmacy, Uppsala University, Uppsala, Sweden;
[4]Department of Clinical Pharmacy, Institute of Pharmacy, University of Hamburg, Hamburg, Germany;
[5]Research, Clinical Pharmacology Modelling & Simulation, GlaxoSmithKline, London, UK;
[6]Department of Biology, Ludwig-Maximilians University Munich, Martinsried, Germany;
[7]German Center for Diabetes Research (DZD e.V.), Neuherberg, Germany;
[8] Department of Pharmacy, Radboud institute of health sciences, Radboud university medical center, Nijmegen, The Netherlands

[¥] These authors contributed equally

*Correspondence: Ulrika.Simonsson@farmbio.uu.se, Department of Pharmaceutical Biosciences, Uppsala University, Box 591, 75124 Uppsala, Sweden

Supplementary Table S1

Table S1. Final model hyperparameters for the different machine learning models.

Algorithm	Hyperparameter	Hyperparameter interval	Hyperparameter values AUC models	Hyperparameter values longitudinal models
**LASSO**	Lambda	(5:20, steps = 1)	5	5
**Random Forest**	Mtry	(1:20, steps = 1)	6	9
**Gradient Boosting Machine (GBM)**	n.trees	(100, 150, 200, 250, 300, 350, 400)	350	200
	Interaction.depth	(1:18, steps = 1)	1	5
	Shrinkage	(0.01, 0.1, 0.9)	0.01	0.1
	n.minobsinnode	(0.005, 0.01, 0.1, 0.5, 1, 5, 10, 20)	0.01	5
**Extreme Gradient Boosting (XGBoost)**	Nrounds	(200:1000, steps = 50)	650	1000
	Eta	(0.005, 0.01, 0.015, 0.025, 0.05, 0.1)	0.005	0.01
	Max_depth	(1, 2, 3, 4, 5, 6, 10)	3	4
	Gamma	(0, 0.05, 0.1, 0.5, 0.7, 0.9, 1.0)	0.5	0.9
	Colsample_bytree	(0.4, 0.6, 0.8, 1)	1	0.8
	Min_child_weight	(1:20, steps = 1)	3	8
	Subsample	(0.1, 0.2, 0.5, 0.75, 1)	0. 2	0.5

Colsample_bytree, subsample ratio of columns when constructing each tree; *eta*, scales the contribution of each tree by a factor (between 0 an 1); *gamma*, minimum loss reduction required to make a further partition on a leaf node of the tree; *interaction.depth*, maximum depth of each tree; *max_depth*, maximum tree depth; *min_child_weight*, minimum number of instances needed in each node; *mtry*, number of variables randomly sampled as candidates at each split; *n-minobsinnode*, minimum number of observations in the terminal nodes of the trees; *nrounds*, maximum number of iterations; *n.trees*, total number of trees to fit; *shrinkage*, learning rate or step-size reduction; *subsample*, subsample ratio of the training data used priot to growing trees.
